# miRNA-Based Breast Cancer Subtyping Using AHALA Multi-Stage Classification Approach

**DOI:** 10.3390/cancers18040586

**Published:** 2026-02-10

**Authors:** Mohammed Qaraad, Eric P. Rahrmann, David Guinovart

**Affiliations:** The Hormel Institute, University of Minnesota, 801 16th Ave NE, Austin, MN 55912, USA; qaraa001@umn.edu (M.Q.); rahr0003@umn.edu (E.P.R.)

**Keywords:** miRNA multi-classification, breast cancer subtyping, optimization algorithms, miRNA biomarkers, neural network

## Abstract

Background: Breast cancers are heterogeneous in nature, including many molecular subtypes, each displaying varying characteristics in clinical outcomes as well as in responses to treatments. Subtyping requires absolute precision for the application of precision medicine; however, this is not an easy task, given the dimensionality as well as noise in miRNA expression profiles. Even though miRNAs display potential as a biological marker for subtyping breast cancers, feature selection and optimizing learning algorithms would help harness their potential as a diagnostic tool. Methods: We propose the Adaptive Hill Climbing Artificial Lemming Algorithm (AHALA), a hybrid optimization framework that integrates the global search capability of the Artificial Lemming Algorithm with an adaptive hill-climbing local search strategy. Low-variance filtering and differential gene expression analysis were first applied to reduce dimensionality and enhance biological relevance. AHALA was then used to optimize deep neural network hyperparameters for miRNA-based multi-class breast cancer subtype classification. The method was validated using TCGA breast cancer miRNA expression data and benchmarked against state-of-the-art optimization algorithms using the CEC2021 test suite. Results: AHALA had a high classification performance measure for each type of breast cancer with a mean accuracy of 95.74%, precision of 95.98%, recall of 95.74%, F1 measure of 95.74%, and AUC value of 0.9682. The new algorithm had superior convergence and significance compared with other optimization algorithms. Feature selection revealed miRNAs that belong to each subtype, such as hsa-miR-190b, hsa-miR-429, hsa-miR-505-3p, hsa-miR-3614-5p, and hsa-miR-935. Conclusions: The AHALA framework offers a potent and efficient method of performing miRNA-based subtyping of breast cancer that integrates global exploration and local search to its advantage. Its high level of classification, stability, and ability to identify biologically important biomarkers mark this method as promising.

## Simple Summary

Breast cancer is a non-homogeneous disease and consists of diverse molecular subtypes that vary based on their prognosis and treatment response. Subtype classification of breast cancer is a crucial step toward improving diagnostic efficiency and personalized treatment. MicroRNAs (miRNAs) are a group of small regulatory RNAs that have been shown to possess great promise as a biomarker for classifying cancers. But analyzing data related to miRNAs is a challenge due to the complexities involved. In this work, we proposed an optimization algorithm dubbed the Adaptive Hill Climbing Artificial Lemming Algorithm (AHALA), which is designed to improve miRNA subtyping in breast cancers. Our proposed algorithm combined feature selection based on biological knowledge with the use of a machine learning algorithm in order to uncover the important miRNAs. Using publicly available datasets of breast cancers, the proposed algorithm showed promising results in distinguishing subtypes of breast cancers, as well as identifying important miRNAs as breast cancer subtyping biomarkers.

## 1. Introduction

Breast cancer is a highly heterogeneous disease that is commonly classified into four molecular subtypes based on the expression levels of hormone receptors and human epidermal growth factor receptor 2 (HER2): Luminal A, Luminal B, HER2-enriched, and Basal-like subtypes [1]. Each subtype exhibits distinct clinical outcomes, prognoses, and therapeutic responses [2]. Luminal A tumors, which are generally hormone receptor-positive and HER2-negative, tend to grow more slowly and are associated with a favorable prognosis, making them highly responsive to hormone therapies such as tamoxifen or aromatase inhibitors [3]. Luminal B tumors, while also hormone receptor-positive, demonstrate higher proliferation rates, often necessitating more aggressive treatment regimens, including chemotherapy in addition to hormone therapy [4]. HER2-enriched breast cancers, characterized by the overexpression of the HER2 protein and typically lacking hormone receptors, are more aggressive but respond well to HER2-targeted treatments such as trastuzumab, which has significantly improved patient outcomes [5]. On the other hand, Basal-like breast cancers, which show gene expression patterns resembling basal epithelial cells and lack both hormone receptors and HER2 expression, represent a highly aggressive and heterogeneous subtype. Chemotherapy remains the primary treatment option for this group due to the absence of targeted therapies [6]. Identifying and understanding these molecular subtypes is crucial for developing personalized treatment strategies. Ongoing research aims to refine these classifications further and develop novel therapeutic approaches to improve patient outcomes across all breast cancer subtypes. The roles of microRNAs (miRNAs) in breast cancer biology are both significant and intricate. miRNAs are short non-coding RNAs that regulate gene expression and are implicated in various biological processes, including cancer development and progression.

Several studies have indicated that miRNAs hold great promise as potential biomarkers for cancer diagnosis, prognosis, and therapy [7,8,9]. Identifying these miRNA biomarkers is crucial due to their role in regulating gene expression, with their dysregulation being a common feature in many cancers. In cancerous cells, miRNAs can be overexpressed, acting like oncogenes by down-regulating tumor suppressor genes, which ordinarily work to prevent uncontrolled cell growth. Conversely, miRNAs that generally act as tumor suppressors may be underexpressed, leading to the upregulation of oncogenes, further driving cancer progression [10]. The dual role of miRNAs in both upregulation and downregulation makes them valuable as biomarkers for cancer. However, identifying specific miRNAs as biomarkers is complex, primarily due to the heterogeneous nature of cancer and the high dimensionality and noise present in miRNA expression datasets [11,12].

Numerous studies have focused on identifying miRNAs associated with breast cancer using a variety of datasets and methodological approaches, particularly in feature selection. Standard methods include Information Gain, Chi-Squared [13], and LASSO, combined with classification models like Random Forests and Support Vector Machines [14]. Despite progress, many of these studies have limitations, as they often focus only on miRNAs discovered through wet lab experiments, potentially overlooking recently identified miRNAs that could be effective biomarkers [15]. Various ensemble feature selection techniques have been explored, including Stochastic Gradient Descent (SGD), Support Vector Machine classifier (SVC), gradient boosting, random forest, logistic regression, passive-aggressive classifier, ridge classifier, and bagging [16]. Other methods involve ANOVA, Mutual Information, Extra Trees Classifier, and Logistic Regression (LGR) [15], genetic algorithms [17], and LASSO [18]. However, much of this work is restricted to binary classification, such as distinguishing Triple-Negative Breast Cancer (TNBC) from non-TNBC [16], breast cancer from normal tissue [15,17], or breast cancer from other cancer types [18].

Moreover, some identified miRNAs, such as those reported in ref. [15], have not undergone validation. Without enrichment analysis, there is a higher risk of false positives—miRNAs that may be statistically significant but lack biological relevance to breast cancer. Enrichment analysis is essential for filtering out such false discoveries, ensuring the identified miRNAs are biologically meaningful and relevant. Among existing studies, only a few [14,19] have focused on the multi-class classification of breast cancer subtypes. In ref. [14], miRNAs were first filtered using XGBoost and Random Forest, and then the mutual information between molecules was calculated and used as a threshold in multilayer network analysis. However, the method to determine this threshold was overly complex, and no enrichment analysis was conducted. Similarly, the feature selection method in ref. [19] was intricate, involving eight mutual information-based algorithms, such as MIM, mRMR, CMIM, JMI, DISR, ICAP, CIFE, and CONDRED. The feature subsets from each algorithm were carefully combined and used with the Random Forest classifier, resulting in a complicated but effective selection process. However, these methods are binary classification-centric, disregarding the multi-class aspect of breast cancer subtyping, and may lack biological verification by enrichment analysis, which may result in false positives [14,19].

To overcome these difficulties, hybrid optimization approaches integrating global and local search strategies have been developed as effective solutions for feature selection in high-throughput omics datasets. Hybrid algorithms integrating Adaptive Hill Climbing or its variants (e.g., Adaptive Beta Hill Climbing, Late Acceptance Hill Climbing) with metaheuristic strategies such as Genetic Algorithms (GAs), Particle Swarm Optimization (PSO), and bio-inspired algorithms have been promising in the area of bioinformatics [20,21,22]. For instance, a kSHAP-ABHC hybrid of Game Shapley Additive exPlanations and Adaptive Beta Hill Climbing achieved 99.9% accuracy for RNA-seq-based cancer classification, i.e., breast cancer, via the optimization of gene subsets [20]. Similarly, a Red Fox Optimization and Dynamic Mutation Late Acceptance Hill Climbing hybrid detected metabolomic biomarkers for lung cancer with an AUC of 0.9926 [21]. Adaptive Beta Hill Climbing has also been hybridized with metaheuristics such as Whale Optimization for gene selection in microarray data, with 100% accuracy in the minimum number of genes [22]. Such hybrids as Genetic Algorithm with Hill Climbing or Harris Hawk Optimization with β-Hill Climbing have also been used for feature selection in various applications, from voxel selection in neuroimaging to dataset-wide feature selection [23,24].

These hybrid approaches possess several advantages. They balance global exploration and local exploitation, coping with complex search spaces to select discriminative features, enhance classification accuracy, and manage high-dimensional, noisy data [20,21,22]. For example, kSHAP-ABHC’s robustness over multiple cancer types and DM-LAHC’s dynamic mutation provide local search adaptability [20,21]. However, these approaches have drawbacks. The high computational cost of models such as kSHAP or GA-based hybrids can restrict scalability to big omics datasets [20,23]. Parameter tuning, i.e., mutation rates for DM-LAHC or velocity updates for PSO-based hybrids, can be challenging and result in unstable performance [21,24]. Moreover, none of these approaches offer adaptive strategies to dynamically optimize exploration vs. exploitation, as needed for heterogeneous miRNA datasets.

Inspired by these limitations, we propose a refined methodology to improve feature selection and breast cancer subtyping. Instead of employing association rule mining, we begin by removing low-variance genes from the dataset. The rationale behind this step is that genes with low variance across samples are less likely to be informative for classification, as they do not exhibit significant changes in expression that could distinguish between different cancer subtypes. Eliminating such genes reduces the dimensionality of the data and improves the overall efficiency of subsequent analyses. Following this, we apply differential gene expression (DGE) [25] analysis to identify genes that are significantly differentially expressed between breast cancer subtypes. DGE allows us to pinpoint biomarkers that may play crucial roles in distinguishing between subtypes based on their expression profiles, thereby enhancing the biological relevance of our feature set. This step is critical as it helps focus the classification task on the most biologically significant genes, rather than including a broad range of potentially irrelevant features. Once the key genes are selected, we introduce the Adaptive Hill Climbing Artificial Lemming Algorithm (AHALA). This new hybrid optimization approach weaves the bio-inspired Artificial Lemming Algorithm (ALA) and Adaptive Hill Climbing. ALA, driven by lemming behaviors such as migration and burrowing, excels at global exploration of high-dimensional search spaces [26]. Its exploitation step, however, can be upgraded towards achieving faster convergence and solution quality. Integrating Adaptive Hill Climbing with solution refinement at regular intervals and dynamic step-size reduction allows AHALA to avoid parameter sensitivity and computational demands of current hybrids while guaranteeing robust feature selection and neural network optimization. AHALA is designed for breast cancer subtyping based on miRNA, where it identifies biologically relevant biomarkers and records high classification accuracy for Luminal A, Luminal B, HER2-enriched, and Basal-like subtypes. This study utilizes a public TCGA dataset that contains miRNA expression profiles and clinical data to validate AHALA’s performance [14]. The main contributions of this work include:

• Development of AHALA, a novel hybrid optimization algorithm that balances global exploration and local exploitation for improved feature selection and classification.
• AHALA application to breast cancer subtyping: Fine-tuning a deep neural network to classify breast cancer subtypes using differentially expressed genes accurately.
• Key biomarker identification: Utilizing AHALA to pinpoint critical miRNAs and genes associated with breast cancer subtypes, offering potential diagnostic and therapeutic targets.
• Validation on CEC2021 benchmark: Demonstrating AHALA’s versatility and effectiveness as a general-purpose optimization technique for diverse, complex challenges.

Through extensive experimentation on breast cancer datasets, AHALA significantly improves classification accuracy, precision, recall, and F1-score, highlighting its potential as a powerful tool in breast cancer subtyping and biomarker discovery. The remainder of this paper is organized as follows: Section 2 details the methodology and data used in our study, Section 3 presents the results, Section 4 discusses the biological significance of our findings, and Section 5 concludes with future directions for research.

## 2. Methodologies

In this section, we outline the methodologies employed, beginning with an overview of the Artificial Lemming Algorithm (ALA) process, followed by a detailed description of the proposed Adaptive Hill Climbing Artificial Lemming Algorithm (AHALA) and its enhancements for optimization tasks.

### 2.1. Artificial Lemming Algorithm (ALA)

Arctic ecosystems are home to a remarkable micromammal known as the lemming, a member of the Cricetidae family. These small rodents, typically measuring around 10 cm in length, are primarily found in tundra, forest-tundra, and mountain-tundra habitats [26]. Lemmings exhibit a distinctive morphology, characterized by a rotund body shape and pelage that varies in coloration depending on the species, ranging from grey to yellow and brown hues. One of the lemmings’ most notable anatomical features is a specialized flattened claw on the first digit of their anterior appendages, which aids in excavating snow-covered terrain. Their dietary habits are predominantly herbivorous, with a preference for graminoids and bryophytes. However, they display dietary flexibility when necessary, consuming foliage, fruits, bulbous structures, roots, and even lichens found atop the snow layer [27]. Within the Arctic food web, lemmings occupy a precarious position, facing constant predation pressure from various carnivores, including Bubo scandiacus (snowy owls), mustelids (weasels), and Ursus maritimus (polar bears). Despite these challenges, lemmings have evolved an extraordinary reproductive capacity. They can produce up to eight litters annually, with each litter potentially yielding up to a dozen offspring. Furthermore, their sexual maturation occurs rapidly, with individuals attaining reproductive capability within a mere 20 to 30 days post-partum [28]. To support their prodigious reproductive output, lemmings have developed the ability to consume quantities of food equivalent to twice their body mass in a single feeding session. Their annual behavioral patterns include springtime movements to higher elevations, where they inhabit mountainous areas and forested regions. During this period, they engage in continuous reproduction before returning to their tundra habitats in autumn [29]. Lemmings rely heavily on their acute olfactory and auditory senses to locate sustenance both above and below ground. When confronted with predators, they employ a diverse repertoire of evasive strategies. As skilled fossorial animals, lemmings construct intricate subterranean networks of tunnels and chambers, which serve dual purposes as secure dwellings and food storage facilities [30]. The ALA algorithm’s mathematical model is presented as follows:

#### 2.1.1. Initialization

The Artificial Lemming Algorithm (ALA) is a population-based optimization method that begins by initializing the positions of search agents. These initial candidate solutions form a matrix Z, with N rows (population size) and Dim columns (number of dimensions), constrained by the problem’s upper and lower bounds, as outlined in Equation (1). The best solution found during each iteration is considered the optimal or near-optimal result at that stage. Decision variables $z_{i,j}$ for each dimension are calculated using Equation (2).

(1) $$Z=\left[\begin{array}{cccc} z_{1,1} & z_{1,2} & \ldots & z_{1,dim}\\ z_{2,1} & \ddots & \ddots & z_{2,dim}\\ \vdots & \ddots & \ddots & \vdots \\ z_{n,1} & z_{n,2} & \ldots & z_{n,dim} \end{array}\right],$$

(2) $$z_{i,j}={ub}_{j}+rand\times \left({ub}_{j}-{lb}_{j}\right).$$

In the Artificial Lemming Algorithm (ALA), the random value rand falls within the range [0,1]. The variables ${lb}_{j}$ and ${ub}_{j}$ represent the lower and upper bounds, respectively, for the $j^{th}$ dimension. These values are used to calculate the decision variables $z_{i,j}$, which define the positions of the search agents within the specified limits of each dimension.

#### 2.1.2. Long-Distance Migration (Exploration)

In the first behavioral phase of the Artificial Lemming Algorithm (ALA), lemmings randomly perform long-distance migrations when food becomes scarce due to overpopulation. During this exploration phase, lemmings move through the search space, guided by their current position and the positions of random individuals within the population. This migration aims to discover regions with richer resources and improved living conditions. Notably, the direction and distance of the migration vary depending on ecological factors, making the movement dynamic. This behavior is mathematically modeled by the following equation:

(3) $$z_{t+1}=z_{best}\left(t\right)+F\times BM\times \left(\begin{array}{c} R\times \left(z_{best}\left(t\right)-z_{i}\left(t\right)\right)\;+\\ \left(1-R\right)\times \left(z_{i}\left(t\right)-z_{a}\left(t\right)\right) \end{array}\right)$$

In this model, $z_{t+1}$ represents the updated position of the $i^{th}$ search agent at iteration(t + 1), while $z_{best}\left(t\right)$ indicates the best solution found so far. The variable F, introduced in Equation (5), serves as a flag that alters the search direction to prevent the algorithm from getting stuck in local optima, thus ensuring more comprehensive exploration of the search space. BM refers to a vector of random numbers simulating Brownian motion, which introduces dynamic and uniform step sizes, helping the search agents explore potential regions more effectively. The step size for Brownian motion is determined by the probability density function of a normal distribution with a mean of 0 and variance of 1, as defined in Equation (4). Additionally, R is a vector of size 1×Dim whose elements are random values uniformly distributed within the range [−1, 1], generated using Equation (6).

(4) $$f_{BM}\left(x;0,1\right)=\frac{1}{\sqrt{2\pi }}\times exp\left(-\frac{x^{2}}{2}\right),$$

(5) $$F=\left\{\begin{array}{c} 1\;\;\;\;if\;\left|2\times rand+1\right|=1\\ -1\;if\;\left|2\times rand+1\right|=2 \end{array}\right.,$$

(6) $$R=2\times rand\left(1,Dim\right)-1.$$

#### 2.1.3. Digging Holes (Exploration)

The second behavior in the Artificial Lemming Algorithm (ALA) models lemmings digging burrows in their environment, forming intricate tunnel networks for protection and food storage. Lemmings create new burrows randomly, guided by their current position and the locations of other random individuals in the population. This behavior enhances their ability to evade predators and search for food more efficiently. The digging process is mathematically modeled by Equation (7).

(7) $$z_{i}\left(t+1\right)=z_{i}\left(t\right)+F\times L\times (z_{best}\left(t\right)-z_{b}\left(t\right)).$$

In this context, L represents a random number related to the current iteration count, influencing the interactions between lemming individuals during the burrow-digging process. $z_{b}$ denotes a randomly selected individual from the population, with being a randomly chosen integer index between 1 and N (population size). These variables L and $z_{b}$ model the cooperative behavior of lemmings as they dig new burrows. The value of L is calculated according to the following equation:

(8) L=rand×(1+sin(t2)),

where t represents the current iteration.

#### 2.1.4. Foraging for Food (Exploitation)

In the third behavior, lemmings engage in extensive, random movements within their burrow networks, utilizing their acute senses of smell and hearing to detect food. They typically confine their foraging efforts to a relatively small area based on the availability and abundance of food. To maximize their intake, lemmings wander randomly within these foraging zones. This stage of behavior is modeled using a spiral wrapping mechanism, which simulates the lemmings’ movement pattern as follows:

(9) $$z_{i}\left(t+1\right)=z_{best}\left(t\right)+F\times spiral\times rand\times z_{i}\left(t\right).$$

In this model, the term “spiral” represents the spiral-shaped trajectory of the lemmings’ random search during foraging. This pattern simulates their movement as they search for food within a limited area. The spiral movement is mathematically described by Equations (10) and (11), which govern the shape and behavior of the foraging process.

(10) $$spiral=radius\times \left(\sin\left(2\times \pi \times rand\right)+\cos\left(2\times \pi \times rand\right)\right),$$

(11) $$radius=\sqrt{\sum {\left(z_{best}\left(t\right)-z_{i}\left(t\right)\right)}^{2}}.$$

In this context, “radius” refers to the radius of the lemmings’ foraging area, defined as the Euclidean distance between their current position and the optimal solution. This radius helps determine the extent of the search area during foraging, influencing the lemmings’ ability to locate food sources effectively.

#### 2.1.5. Evading Natural Predators (Exploitation)

In the final stage, the model emphasizes the avoidance and protective behaviors of lemmings in the face of danger. The burrow acts as a safe refuge for these animals. Upon detecting a predator, lemmings utilize their remarkable running speed to retreat quickly to their burrow. Additionally, they employ deceptive maneuvers to evade capture during pursuit. This behavior is mathematically represented by Equation (12).

(12) $$z_{i}\left(t+1\right)=z_{best}\left(t\right)+F\times G\times Levy(Dim)\times (z_{best}\left(t\right)-z_{b}\left(t\right)),$$

(13) $$G=2\times \left(1-\frac{t}{T_{max}}\right).$$

In this context, G denotes the escape coefficient of lemmings, which quantifies their ability to flee and diminishes as the iteration count increases, as indicated in Equation (13). $T_{max}$ represents the maximum number of iterations for the algorithm. The Lévy flight function, denoted as Levy(z), is utilized to model the deceptive maneuvers lemmings execute while escaping predators. This function is expressed as follows, where *u* and *ν* are random values within the interval [0,1], β is a constant set to 1.5, and z represents the current position.

(14) Levyz=0.01×u×σ|v|1β,σ=(Γ(1+β)×sin(πβ2)Γ(1+β2)×β×2(β−12))1β.

In the Artificial Lemming Algorithm (ALA), the four search strategies are directly linked to the energy levels of the lemmings. During the initial phases, lemmings primarily focus on exploration to identify potential areas. As the search progresses, they shift towards local exploitation to refine their solutions. To achieve a balanced approach between exploration and exploitation, an energy factor is introduced, which decreases over successive iterations. When lemmings possess adequate energy, they actively engage in migration or burrow digging; conversely, when energy is low, they prioritize foraging and evading predators. The formula for calculating the energy factor is as follows:

(15) $$E\left(t\right)=4\times \left(1-\frac{t}{T_{max}}\right)\times In\left(\frac{1}{rand}\right),$$

t represents the current iteration, $T_{max}$ denotes the maximum number of iterations, and rand represents a random number in the range [0,1].

## 3. Proposed Adaptive Hill Climbing Artificial Lemming Algorithm AHALA

The Artificial Lemming Algorithm (ALA) is a bionic meta-heuristic optimization technique inspired by the behavior of lemmings. While ALA has shown promise in solving complex optimization problems, there is potential for improvement in its convergence speed and solution quality. Meta-heuristic algorithms often face challenges balancing exploration (searching global space) and exploitation (refining local solutions). ALA’s strength lies primarily in its exploration capabilities, but it may benefit from enhanced local search mechanisms to improve its exploitation phase.

### 3.1. Insight into Enhancement

The proposed enhancement introduces a local search component, specifically a hill-climbing algorithm, to complement ALA’s global search strategy. This hybridization aims to leverage the strengths of both approaches:

1. ALA’s global search capability helps to explore the solution space broadly and avoid getting trapped in local optima.
2. The hill-climbing algorithm provides a mechanism for fine-tuning promising solutions, potentially accelerating convergence and improving the quality of final solutions.

The selection of Adaptive Hill Climbing as part of AHALA is driven by its local exploitation power, which is complementary to ALA’s global exploratory power. Hill Climbing is a straightforward but effective optimization method that constructs a solution step by step by searching in neighboring states, which is computationally effective in terms of feature subset optimization and neural network hyperparameter optimization [19]. In our research environment, the TCGA miRNA expression dataset contains 231 samples and an initial feature set of 588 miRNAs, narrowed down to 208 following low-variance filtering and differential gene expression (DGE) analysis (Section 5.4.2).

The moderately small dataset size is appropriate for Hill Climbing as it prevents the scalability problem faced in vast genomic datasets (e.g., whole-genome sequencing with millions of features).

Classic Hill Climbing can be restricted by being prone to local optima and without global exploration. To address this, AHALA combines Adaptive Hill Climbing with ALA, taking advantage of ALA’s bio-inspired features of long-range migration and burrow digging to explore new areas across the search space. Adaptive Hill Climbing lowers the convergence iterations by employing periodic solution refinement and dynamic step-size decay (parameters α, β) for increased efficiency. The hybrid method retains high classification performance while reducing ALA’s global search computational overhead. Moreover, the pretreatment steps, such as DGE analysis and low-variance filtering, reduce the dimensionality of the miRNA data so that Hill Climbing can be conducted within a tractable feature space. This renders it easier to use and scalable to medical data.

### 3.2. Mathematical Description

Let ${f:R}^{n}\to R$ be the objective function to be minimized, where n is the dimension of the problem space. The enhanced AHA (Adaptive Hill Climbing Artificial Lemming Algorithm) can be described as follows:

Initialization: Initialize a population of N solutions: $X=\left\{x_{1},x_{2},\ldots .,x_{n}\right\}$, where each $x_{n}$∈ $R^{n}$.

Main Iteration Loop: For each iteration t=1,2,…,T

a. Apply the standard AHA update rules to generate new candidate solutions.

b. Evaluate the fitness of the new solutions: $f(x_{i})$ for each $x_{i}$∈ X.

c. Update the best-known solution $x_{best}$

d. If t mod k=0 (where k is a predefined frequency), apply Hill Climbing to $x_{best}$.

Hill Climbing Procedure: For j=1,2,…,M (where M is the maximum number of Hill Climbing iterations):

a. Generate a perturbation vector $\delta_{j}\in R^{n}$, where each component is drawn from a uniform distribution: $\delta_{j}\sim U(-1,1)$

b. Create a new candidate solution:

(16) $$x_{new}=x_{best}+\alpha \delta_{j},$$

c. If $f\left(x_{new}\right)<f(x_{best})$, update $x_{best}=x_{new}$.

d. Otherwise, reduce the step size:

(17) α=βα,

where α is the step size, and β denotes a decay factor 0<β<1.

The parameter k represents the frequency of applying the Hill Climbing procedure, such as every 10 iterations of the AHA algorithm. The maximum number of Hill Climbing iterations is denoted by M, α represents the initial step size for Hill Climbing, and β refers to the step size decay factor. The choice of k plays a critical role in balancing exploration and exploitation. If k is too small (i.e., Hill Climbing is applied too frequently), the algorithm may focus too heavily on local exploitation, potentially neglecting the broader search space and leading to premature convergence. On the other hand, if k is too large (i.e., Hill Climbing is applied infrequently), the algorithm might miss opportunities to refine the best solution found so far, slowing down convergence.

Similarly, the initial step size α and the decay factor β are crucial in controlling the granularity of local search during Hill Climbing. A larger α allows for more significant perturbations, encouraging broader exploration within the neighborhood of the current best solution. However, if α is too large, the algorithm might overshoot optimal solutions. The decay factor β gradually reduces the step size over time, allowing the search to focus more finely on local optima as the algorithm progresses. This decaying step size ensures that, as the search continues, the adjustments become more precise, improving the likelihood of finding a near-optimal solution. Together, these parameters provide flexibility, enabling the algorithm to fine-tune the balance between global search and local refinement, which is essential for efficiently navigating complex, high-dimensional search spaces.

### 3.3. Complexity Analysis

The computational complexity of the Adaptive Hill Climbing Artificial Lemming Algorithm (AHALA) can be understood by analyzing both the standard AHA operations and the additional computational load introduced by the Hill Climbing mechanism.

#### 3.3.1. Standard AHA Complexity

The standard AHA optimization process involves maintaining a population of N candidate solutions, each residing in an n−dimensional space. The algorithm iterates over a total of T iterations, and the computational cost of evaluating the objective function f for each candidate. The solution is denoted by O(E), where E represents the cost of a single fitness evaluation. Thus, the total time complexity for the standard AHA algorithm can be expressed as: O(NTnE) This term captures the cost of evaluating all candidate solutions over T iterations across n−dimensional problem space.

#### 3.3.2. Hill Climbing Enhancement Complexity

The integration of the Hill Climbing mechanism into AHA introduces additional computational costs. Hill Climbing is applied periodically, once every k iterations, to refine the best-known solution $x_{best}$. For each application of Hill Climbing, a maximum of M iterations are performed, where each iteration requires the generation of a perturbation vector in the n−dimensional space and a fitness evaluation. The computational complexity of the Hill Climbing phase per application is thus O(MnE), and since Hill Climbing is applied $\frac{T}{K}$ times during the entire optimization process, the total additional complexity is given by:

(18) $$O\left(\frac{T}{K}MnE\right).$$

#### 3.3.3. Overall Complexity

Combining the time complexity of the standard AHA and the additional cost introduced by Hill Climbing, the overall time complexity of the AHALA can be expressed as:

(19) $$O\left(TnE\left(N+\frac{M}{k}\right)\right).$$

This expression indicates that the computational load grows linearly with the number of iterations T, the dimensionality of the problem n, and the cost of evaluating the fitness function E. The terms N (population size) and $\frac{M}{k}$ (related to the frequency and intensity of Hill Climbing) dictate the relative contributions of global exploration and local exploitation to the overall complexity.

#### 3.3.4. Space Complexity

The space complexity of AHALA remains largely unchanged from the standard AHA algorithm. Since the algorithm only requires storage for the population of candidate solutions and a constant number of variables used for Hill Climbing, the overall space complexity is O(Nn). This space complexity is proportional to the number of candidate solutions N and the dimensionality of the search space n, with no significant additional storage required for the hill-climbing process. The proposed enhancement introduces a trade-off between increased computational cost and potential improvements in convergence speed and solution quality. The additional complexity of $O(\frac{T}{K}MnE)$ is generally acceptable if M and $\frac{1}{K}$ are kept reasonably small. By carefully tuning these parameters, the computational overhead remains manageable while still achieving significant gains in performance. The effectiveness of this enhancement may vary depending on the nature of the optimization problem. It is particularly beneficial for problems with the following characteristics:

• Rugged fitness landscapes with many local optima.
• Problems where small, local improvements can significantly impact the overall solution quality.
• Scenarios where the computational budget allows for additional local search steps.

**Figure 1:**
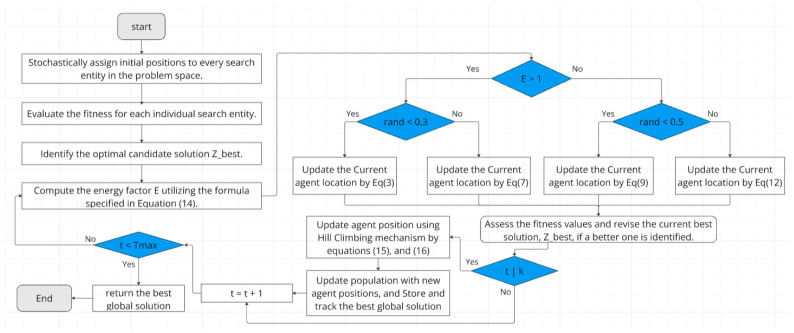
Flowchart of the Adaptive Hill Climbing Artificial Lemming Algorithm (AHALA), illustrating the iterative process of population initialization, exploration (migration and burrow digging), exploitation (foraging and predator evasion), periodic hill climbing for local refinement, and convergence to the optimal solution.

The parameters k, M, α and β provide flexibility in balancing the global exploration of ALA with the local exploitation of Hill Climbing. These parameters can be tuned based on the specific problem characteristics and computational resources available. Future work could explore adaptive mechanisms for automatically adjusting these parameters during the optimization process, potentially leading to a more robust and efficient algorithm across a wider range of problem types. Algorithm 1 presents the pseudocode for the AHALA algorithm, while Figure 1 provides a visual representation of the algorithm’s procedural flow.
**Algorithm 1:** The AHALA**Initialize parameters:** Population size (N), Maximum iterations ($T_{max}$), Dimensions (Dim), Upper and lower bounds (ub, lb) **Initialize exploration parameters:** Frequency of Hill Climbing (k), Maximum Hill Climbing Iterations (M) **Set step size parameters:** Initial Step Size (α), Step size decay factor (β) **Initialize** a population of agents (Z) with random positions within the search space **Evaluate** the fitness of each agent Calculate the Current Optimal Solution $Z_{best}$ **For** (t = 1 to $T_{max}$) Calculate the value of E by Equation (14)
**For** each search agents Z
**If** E > 1 then
**If** rand < 0.3 then Update the current Position by Equation (3)
**else** Update the current Position by Equation (7)
**else**

**If** rand < 0.5 **then** Update the current Position by Equation (9)
**else** Update the current Position by Equation (12)
**End** Evaluate the fitness of all modified population
**If** the current iteration is divisible by k:  Perform Hill Climbing on top-performing agents: 
**For** each selected agent (based on fitness ranking):  Initialize step size = α 
**For** t = 1 to M:  Update agent position using Hill Climbing mechanism by Equation (15) Evaluate the new fitness 
**If** new fitness is better, **accept** the solution  Decay the step size by factor β by Equation (16)
**End** Update population with new agent positions Store and track the best global solution $Z_{best}$ **End** **Return:** Best global solution $Z_{best}$, and it’s fitnes value ${fitness}_{best}$

## 4. Experimental Validation and Performance Analysis

To evaluate the robustness and effectiveness of the proposed Adaptive Hybrid Artificial Lemming Algorithm (AHALA), we conducted comprehensive computational experiments utilizing the CEC2021 benchmark suite. This established benchmark framework provides a rigorous testing environment for assessing optimization algorithms across various complexity levels, offering challenging scenarios that effectively test both established and emerging metaheuristic approaches. To establish a comprehensive performance baseline, we implemented comparative analyses against six contemporary metaheuristic algorithms developed between 2020 and 2024. The comparison group consisted of:

• The RIME algorithm [31].
• Q-learning embedded sine cosine algorithm (QleSCA) [32].
• Enhanced Tug of War Optimization (EnhancedTWO) [33].
• Random Walk Grey Wolf Optimizer (RWGWO) [34].
• Gradient-based optimizer (GBO) [35].
• Standard Artificial Lemming Algorithm (ALA).

For experimental consistency, we maintained uniform initial conditions across all algorithms. The population size (N) was standardized at 30 individuals, with a maximum evaluation threshold of 2500 iterations. Implementation parameters for each comparative algorithm were configured according to their respective original publications, as detailed in Table 1. The experimental framework utilized Python (version 3.8.19) implementations executed on a high-performance computing environment running Rocky Linux 9.4 (Blue Onyx). The hardware configuration comprised an AMD EPYC 7713 64-core Processor(Advanced Micro Devices, Inc., Santa Clara, CA, USA) supported by 1TiB RAM, ensuring consistent computational capabilities throughout the testing phase. To account for the stochastic nature of these algorithms, we executed 30 independent trials for each method. Performance metrics were calculated using multiple statistical indicators:

• Mean fitness values (Avg).
• Standard deviation (std).
• Median performance (Med).
• Friedman rank statistics.
• Wilcoxon signed-rank test results.

### 4.1. Qualitative Analysis

**Figure 2:**
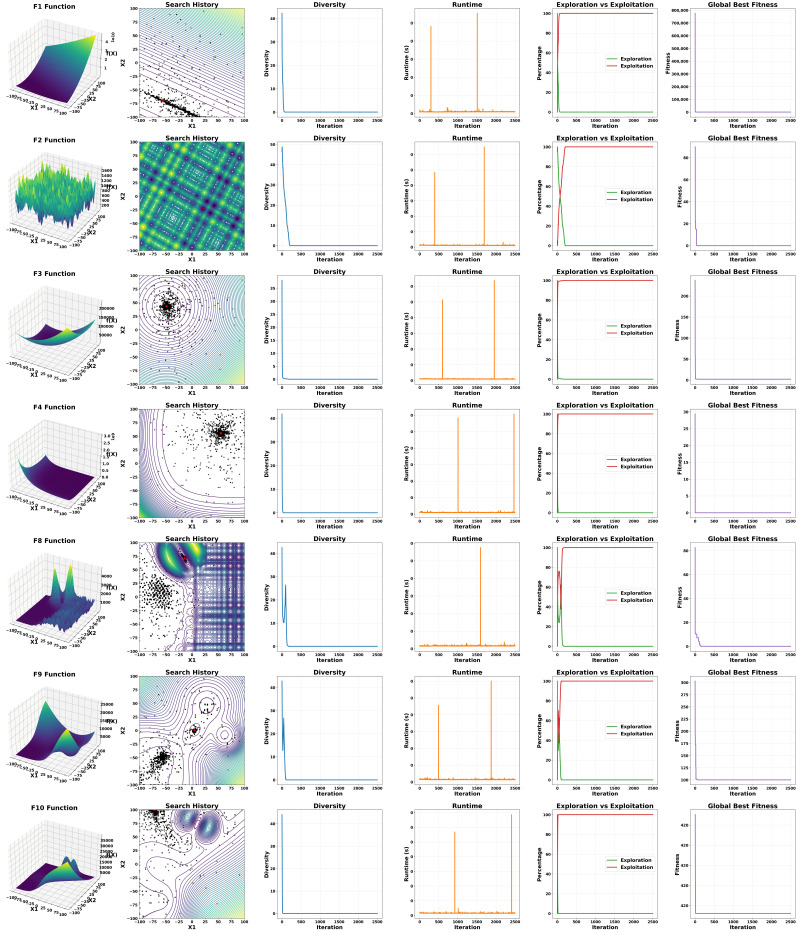
Qualitative analysis of AHALA on six CEC2021 benchmark functions, illustrating 3D surface plots, search history, diversity measurements, runtime behavior, exploration–exploitation balance, and global best fitness over iterations.

In this analysis, the performance of AHALA was evaluated on six CEC2021 benchmark functions (f1, f2, f3, f4, f8, f9, and F10) as shown in Figure 2, each presenting unique optimization challenges. The analysis is based on six key performance indicators: function landscape, search history, diversity measurement, runtime, exploration vs. exploitation balance, and global best fitness.

Function Landscape and Search History: The 3D surface plots and 2D contour plots reveal the diverse nature of the benchmark functions. f1 and f4 appear to be unimodal, while f2, f3, f8, f9, and F10 exhibit multimodal characteristics with varying degrees of complexity. The search history overlaid on the contour plots demonstrates AHALA’s ability to navigate these landscapes effectively. For instance, in f1 and f4, the algorithm converges quickly to the global optimum region, whereas in more complex functions like f8, F9 and f10, it explores multiple promising areas before convergence.

The choice of f1, f2, f3, f4, f8, f9, and f10 was based on their suitability for 2D visualization and application to our study’s optimization tasks. f1, being a unimodal function, offers a smooth surface to evaluate convergence rate, and f2–f4, being multimodal and hybrid functions, evaluate AHALA’s performance in addressing local optima, simulating the non-linear search space of neural network hyperparameters. f8, f9, and F10, as composition functions, are a challenge to AHALA’s global search in complex, deceptive landscapes, revealing its exploration–exploitation trade-off. Hybrid functions f5, f6, and f7 were excluded from qualitative analysis as they are set for larger dimensions (min dim = 10 or 20) within the CEC2021 suite and are therefore incompatible with the 2D case required by our visualization-based analysis. The hybrid nature of f5–f7 relies on shuffle data and matrix operations that are currently only implemented for dim = 10 or 20 and thus are unsuitable for 2D landscape and diversity plots. Nevertheless, f3 and f4 provide good representations of multimodal and hybrid behavior in dim = 2, providing strong qualitative information.

Diversity Measurement: Across all functions, AHALA maintains a relatively high diversity in the initial stages, which gradually decreases as the search progresses. This trend is particularly pronounced in f1 and f4, indicating rapid convergence. In contrast, for multimodal functions like F2 and F10, the diversity reduction is more gradual, suggesting sustained exploration of the search space, which is a key benefit of its adaptive nature and hill-climbing exploitation mechanisms.

Runtime Performance: The runtime charts show sporadic spikes across all functions, which could indicate periodic intensive computations or adaptive mechanisms within AHALA. The frequency and magnitude of these spikes vary across functions, potentially reflecting the algorithm’s responsiveness to different landscape complexities. The relatively consistent runtime across iterations implies that the algorithm is not heavily burdened by significant fluctuations in computational cost, making it suitable for handling high-dimensional problems.

Exploration vs. Exploitation Balance: A common pattern observed across all functions is a high initial exploration phase followed by a transition to exploitation. The transition point and the rate of change vary depending on the function’s complexity. For simpler functions like F1, the transition occurs earlier, while for more complex functions like F10, the exploration phase is prolonged. This adaptive control is crucial in avoiding stagnation in local minima, which can be observed by the balance of exploration and exploitation percentages over iterations.

Global Best Fitness: The convergence behavior of AHALA differs significantly across functions. For F1 and F4, rapid initial improvement is followed by fine-tuning. In contrast, functions like F2 and F10 show more gradual improvement, with occasional significant jumps in fitness, indicative of escaping local optima. The convergence pattern indicates that AHALA is robust against varying problem complexities.

The proposed algorithm AHALA demonstrates adaptive behavior across diverse function landscapes. It effectively balances exploration and exploitation, showing rapid convergence for simpler functions while maintaining the ability to navigate complex, multimodal spaces. The algorithm’s performance on these CEC2021 benchmarks suggests its potential versatility in handling a wide range of optimization problems. However, further quantitative analysis and comparison with other state-of-the-art algorithms would be necessary to fully assess its efficacy and efficiency.

### 4.2. Comparison with Recent Optimizers

The discussion of numerical results highlights the comparative analysis of the proposed AHALA algorithm against six contemporary optimizers using the CEC2021 benchmark suite, with a focus on performance across various optimization scenarios (f1–f10). The findings reveal significant performance differences, as shown in Table 2, where the highlighted (bold) values indicate the best results achieved for each function among the compared algorithms, demonstrating AHALA’s strengths in global optimization and stability across different functions. In the context of global optimization, f1 Function, AHALA shows exceptional performance with an average value of $1.0280\times 10^{2}$, outperforming all competitors by several orders of magnitude. The closest competitor, ALA, achieves $1.4208\times 10^{3}$, while other algorithms, such as QleSCA, present much higher values $1.8437\times 10^{10}$. Statistical validation through the Wilcoxon signed-rank test supports this finding, consistently showing a p-value of $1.86\times 10^{-9}$ when compared to all competitors, indicating that AHALA’s performance is statistically significant at the α = 0.05 level, as shown in Table 3.

The stability and consistency of AHALA are also noteworthy, as demonstrated by its standard deviation metrics across functions. For instance, in f6, AHALA’s standard deviation (2.7704 × 10^1^) is substantially lower than that of other competitors, such as RW_GWO (3.7659 × 10^3^). Similarly, for f7, AHALA’s standard deviation ($1.0371\times 10^{3}$) reflects greater stability when compared to RIME ($6.12191\times 10^{4}$) and RW_GWO ($1.2606\times 10^{5}$), underscoring the algorithm’s reliability in maintaining consistent results. In terms of comparative performance, AHALA showcases clear strengths across f1, f5, f6, and f7, where it significantly outperforms other algorithms. For example, in f1, AHALA’s median value ($1.0089\times 10^{2}$) is drastically better than all other competitors. In contrast, functions like f4 and f9 show competitive but not superior performance, with p-values indicating statistical parity between AHALA, ALA (*p* = 0.427955), and RIME (*p* = 0.776569) for f4, and equivalent performance with ALA in f9 (*p* = 1.000).

The statistical significance analysis further supports AHALA’s dominance in certain functions. For f1, the Wilcoxon signed-rank test reveals consistent superiority with p-values of 1.86×10^−9^ (as shown in Table 3) across all algorithm comparisons. In contrast, for f2, significance levels vary, ranging from *p* = 1.86×10^−9^ against QleSCA to *p* = 0.626346 when compared to GBO. Additionally, AHALA shows statistically significant improvements in F5–F7 with strong p-values across all competitors. Several notable performance patterns emerge from this analysis. AHALA demonstrates consistent optimization efficiency, particularly in complex functions like f1, f5, and f6, where it consistently achieves lower average values. Furthermore, the algorithm strikes a balance between exploration and exploitation, a critical factor in ensuring efficient convergence. AHALA also exhibits convergence stability, with lower standard deviations across most functions, highlighting reliable performance. The fact that median values are consistently close to averages across different functions reflects AHALA’s robust convergence properties.

Despite these strengths, certain areas require further investigation. In particular, AHALA’s performance in f2 shows variability in significant levels, which could indicate areas for improvement. Additionally, while competitive, the algorithm does not demonstrate clear dominance in f4 and f9, warranting further exploration of its behavior in these functions.

**Figure 3:**
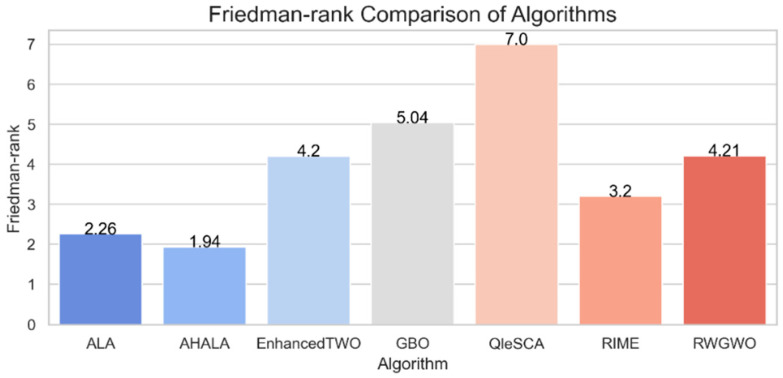
Friedman test results of AHALA and other algorithms on CEC2021.

Figure 3 presents the Friedman test results comparing AHALA with six other optimization algorithms on the CEC2021 benchmark. The Friedman test, a non-parametric statistical test, ranks the algorithms based on their performance across multiple functions. Lower ranks indicate better performance. The proposed AHALA algorithm achieved the second-best average rank (1.94), which demonstrates its competitive edge among the tested algorithms. The Adaptive Local Algorithm (ALA) obtained the best overall rank (2.26), suggesting it performed slightly better than AHALA in terms of consistency. Meanwhile, RIME was ranked third with a Friedman rank of 3.2, followed by EnhancedTWO and RWGWO with ranks of 4.2 and 4.21, respectively. The Gradient-Based Optimizer (GBO) and QleSCA performed less favorably, with GBO receiving a rank of 5.04 and QleSCA the worst rank of 7. These rankings reflect the overall robustness of AHALA across various test functions, positioning it as a highly competitive algorithm among the recent optimization methods.

### 4.3. Convergence Analysis

**Figure 4:**
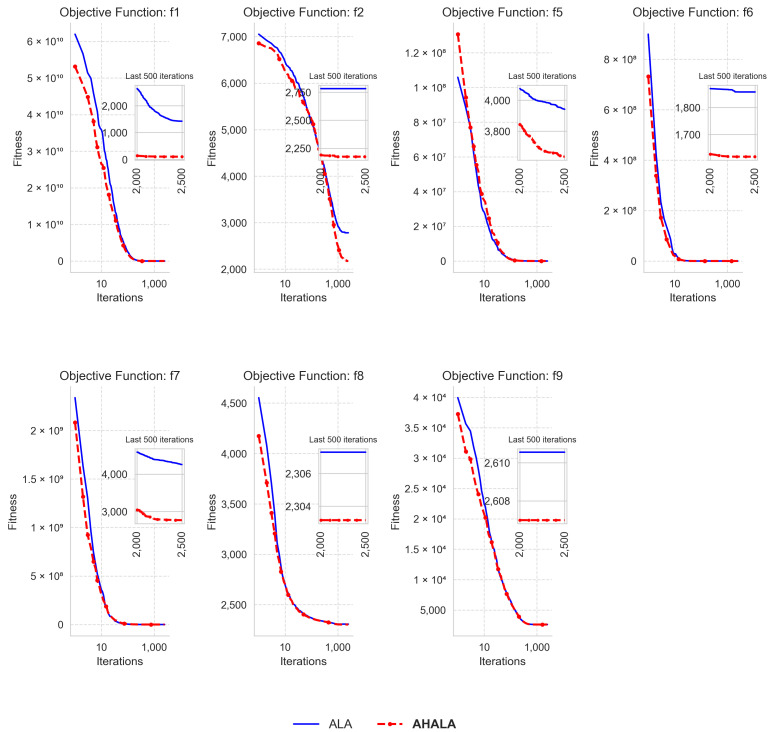
Convergence behavior of AHALA Vs basic ALA on some of the CEC2021 test suites.

In this subsection, we analyze the convergence behavior of the proposed AHALA algorithm in comparison to the basic ALA. Figure 4 illustrates the convergence curves of AHALA and ALA across multiple objective functions from the CEC2021 benchmark. The convergence analysis aims to evaluate the ability of the algorithms to minimize the fitness function over increasing iterations. For objective functions f1, f2, f5, and f7, AHALA demonstrates faster convergence, consistently achieving lower fitness values than ALA, particularly in the later stages of optimization. This indicates AHALA’s superior exploitation capability and its ability to effectively refine solutions as the iterations progress. Notably, for f2 and f5, the gap between AHALA and ALA becomes more pronounced during the final iterations, reflecting AHALA’s enhanced local search mechanism.

In contrast, for functions f6 and f9, AHALA and ALA exhibit similar convergence patterns, with both algorithms achieving comparable fitness values. This suggests that both algorithms are well-suited for optimizing these particular functions, although AHALA retains a slight edge in maintaining a more stable performance towards the end of the search process. The insets within each plot provide further insight into the final 500 iterations, where AHALA maintains a more consistent and lower fitness value across several functions, demonstrating its advantage in fine-tuning solutions during the final optimization phase. The reduced oscillations in the AHALA curve, as seen in functions like f7 and f8, further support its robust convergence behavior. Overall, AHALA outperforms ALA in most of the tested functions, particularly in terms of convergence speed and precision, making it a more effective algorithm for tackling high-dimensional optimization problems.

**Figure 5:**
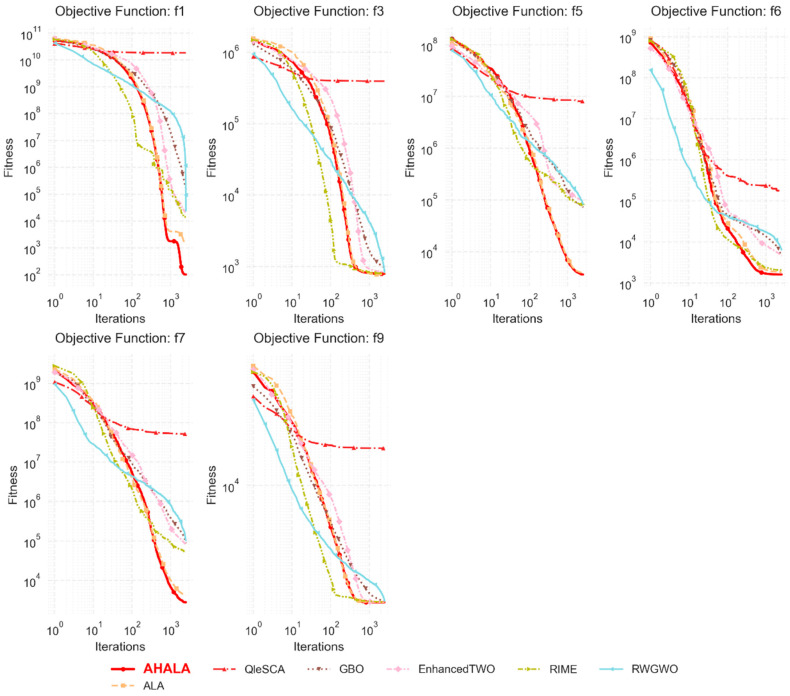
Convergence behavior of AHALA Vs other algorithms on some of the CEC2021 test suites.

On the other hand, the convergence characteristics of the proposed AHALA algorithm were analyzed against six state-of-the-art optimizers across different objective functions from the CEC2021 benchmark suite. Figure 5 illustrates the convergence trends over iterations, providing valuable insights into the algorithmic behavior and optimization efficiency. In function f1, AHALA demonstrates superior convergence characteristics, achieving rapid fitness improvement within the first 100 iterations. The algorithm maintains a consistent descent pattern, ultimately reaching a significantly lower fitness value (approximately 10^2^) compared to competitors like QleSCA, which stagnates around 10^10^. This early-stage efficiency suggests AHALA’s robust exploration capabilities in high-dimensional search spaces. The convergence plots for functions f3 and f5 reveal AHALA’s balanced exploitation–exploration mechanism. In f3, while RIME shows initial rapid convergence, AHALA maintains a steady convergence rate, eventually achieving competitive results around 10^3^ iterations. Similarly, in f5, AHALA demonstrates consistent improvement throughout the optimization process, avoiding premature convergence that is evident in algorithms like QleSCA and Enhanced TWO. Functions f6 and f7 highlight AHALA’s capability for fine-tuning solutions in the later stages of optimization: In f6, AHALA achieves the lowest fitness value (approximately 10^3^) with a smooth convergence curve. For f7, while other algorithms show signs of stagnation after 500 iterations, AHALA continues to improve, reaching superior solutions around 10^3 iterations.

The convergence pattern in f9 is particularly noteworthy, where AHALA maintains stable convergence without the oscillations observed in RWGWO and GBO. This stability is crucial for real-world applications where consistent performance is essential. The algorithm’s ability to avoid local optima is evidenced by its continuous improvement across all test functions, particularly visible in the monotonic descent patterns of f1 and f5. These convergence characteristics validate AHALA’s effectiveness in balancing exploration and exploitation phases, contributing to its robust performance across diverse optimization scenarios. The algorithm’s ability to maintain consistent improvement rates while avoiding premature convergence suggests its potential utility in complex real-world optimization problems.

### 4.4. Boxplot Analysis

**Figure 6:**
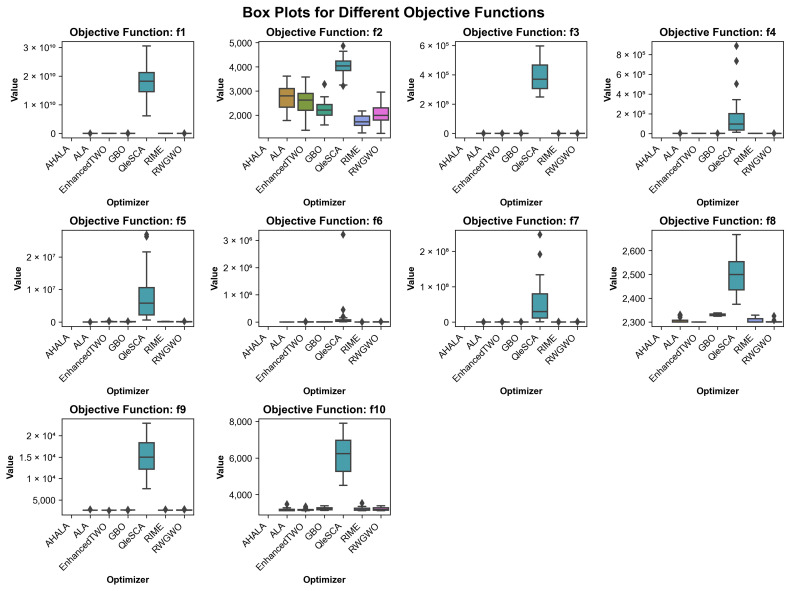
Boxplots of the proposed AHALA and other comparisons on CEC2021 functions.

The comparative performance of the AHALA optimizer against other state-of-the-art algorithms was evaluated across ten objective functions (f1–f10), as illustrated in Figure 6. The box plots reveal several significant patterns in the algorithms’ performance distributions. For most objective functions (f1, f3, f5, f6, f7), AHALA demonstrated remarkable stability, maintaining consistently low objective values with minimal variance, as evidenced by its compact box plots. This stability is particularly notable compared to algorithms like RIME and QleSCA, which often exhibited wider interquartile ranges and more extreme outliers. The superior performance of AHALA is especially pronounced in functions f2 and f4, where it achieved significantly lower median values than other optimizers. In f2, AHALA maintained values consistently below 2000, while competitors like GBO and EnhancedTWO struggled with medians above 2500. Similarly, in f4, AHALA’s compact distribution near the lower bound contrasts sharply with the high variability shown by RINE. Functions f8 through f10 present interesting cases where algorithm performance differentiation becomes more nuanced. In f8, while AHALA maintained competitive performance, algorithms like GBO and QleSCA showed comparable stability. The performance gap between AHALA and other optimizers narrowed in f9 and f10, though AHALA still maintained an edge regarding consistency and median values.

One notable observation is AHALA’s resistance to outliers across all functions, as indicated by the relatively few outlier points in its box plots. This characteristic suggests robust performance across different optimization scenarios, contrasting with algorithms like RIME and EnhancedTWO, which frequently produced extreme outliers, particularly in functions f1, f4, and f7. The box plots also reveal that competing algorithms often exhibited larger interquartile ranges, indicating less predictable performance. This variability is particularly evident in f3 and f5, where algorithms like ALA and GBO showed significant spread in their solution quality, while AHALA maintained tight distributions around optimal values.

## 5. miRNA-Based Subtyping and Classification of Breast Cancer

This section focuses on the use of miRNA data for breast cancer subtyping and classification. It presents a detailed exploration of the proposed methodology, which integrates advanced feature selection techniques with optimization algorithms to identify significant miRNAs for each breast cancer subtype. The aim is to enhance classification accuracy by selecting biologically relevant features and applying an optimized deep neural network classifier to effectively distinguish between the four molecular subtypes of breast cancer. The process is designed to improve both predictive performance and interpretability in cancer diagnostics.

### 5.1. Methodology

**Figure 7:**
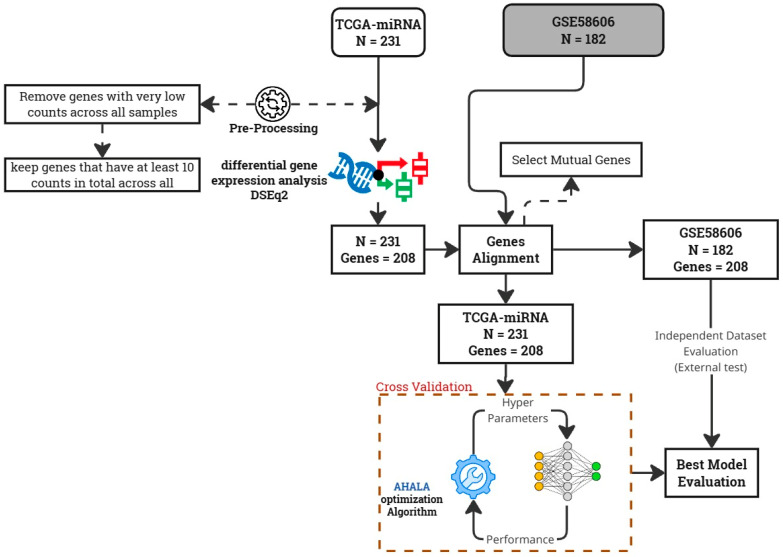
Flowchart of the miRNA-based breast cancer subtyping pipeline using AHALA, depicting the sequential steps of data preprocessing, low-variance gene filtering, differential gene expression analysis, AHALA-driven feature selection, neural network hyperparameter optimization, and subtype classification.

This section provides a detailed account of the methodology implemented in this study. For experimental purposes, data from Breast Invasive Carcinoma (BRCA) were utilized, encompassing four molecular subtypes of breast cancer: Luminal A (LA), Luminal B (LB), HER2-Enriched (H2), and Basal-Like (BL). The dataset comprises 231 samples, including 86 from Luminal A patients, 39 from Luminal B, 24 from HER2-Enriched, 41 from Basal-Like cancer patients, and 41 from Normal samples [14]. The primary aim is to identify key miRNAs associated with each breast cancer subtype through effective feature selection. To achieve this, we introduce a novel methodology designed to enhance both feature selection and classification accuracy in breast cancer subtyping. The methodology is structured to optimize the feature selection process while ensuring the biological relevance of the selected genes. This is followed by the implementation of an advanced optimization algorithm to boost the performance of a deep neural network classifier. The methodological workflow is depicted in Figure 7 and consists of the following stages:

Low-Variance Gene Filtering: We initiate the process by removing genes with low variance across samples from the dataset. The rationale behind this step is that low-variance genes typically do not provide substantial information for classification, as their expression levels remain relatively constant and do not vary significantly between different breast cancer subtypes. By eliminating these genes, we reduce the dimensionality of the dataset, which not only speeds up subsequent analyses but also enhances the focus on more informative and discriminative features.

Differential Gene Expression (DGE) Analysis: After filtering low-variance genes, we apply Differential Gene Expression (DGE) analysis to identify genes that exhibit significant expression differences between the breast cancer subtypes. This analysis is crucial for pinpointing biomarkers that may play important roles in distinguishing between subtypes based on their expression profiles. By focusing on differentially expressed genes, we ensure that the features used for classification are biologically relevant and directly related to the molecular differences between cancer subtypes.

Adaptive Hill Climbing Artificial Lemming Algorithm (AHALA) for DNN Hyperparameter Optimization: After identifying significant genes, the Adaptive Hill Climbing Artificial Lemming Algorithm (AHALA) is employed to optimize the hyperparameters of a deep neural network classifier. Specifically, AHALA optimizes three key hyperparameters: hidden layer size, learning rate, and batch size.

Since the optimization problem involves mixed-type variables, AHALA adopts a hybrid encoding strategy to handle both continuous and discrete parameters within a unified search space. The learning rate is treated as a continuous variable, while the hidden layer size and batch size are modeled as discrete integer variables.

During the optimization process, AHALA internally operates in a continuous search space. Candidate solutions generated for integer-valued hyperparameters are mapped to the nearest valid integer values using a deterministic rounding operator before network training and evaluation. Formally, for a candidate solution x, integer parameters are obtained as:

(20) $$x_{int}=round(x)$$

followed by boundary enforcement to ensure feasibility within predefined limits. The search ranges are defined as follows:

(i) Lower bounds (lb): [0.0001,16,64],
(ii) Upper bounds (ub): [0.01,128,512].

This mixed-variable handling enables AHALA to efficiently explore both integer and continuous dimensions while preserving the structural constraints of the neural network. By integrating adaptive hill climbing mechanisms, the algorithm enhances local refinement around promising regions and mitigates premature convergence, resulting in improved classification accuracy and stability.

### 5.2. Fitness Function Definition

In the proposed AHALA-miRNA framework, the optimization objective is to maximize the classification performance of the deep neural network. Accordingly, the overall classification accuracy on the validation set is employed as the fitness function. For a given candidate solution x, representing a specific configuration of selected features and DNN hyperparameters, the fitness function is defined as:

(21) Fitness(x)=max(Accuracy(x))

During optimization, AHALA iteratively evaluates candidate solutions by training the DNN using the selected miRNA features and corresponding hyperparameters, and then computing the resulting validation accuracy. Candidate solutions yielding higher accuracy values are favored and guide the search process toward optimal regions of the solution space. Accuracy was selected as the fitness function because the primary goal of this study is to achieve correct subtype classification across multiple breast cancer classes. To mitigate the limitations of accuracy in the presence of class imbalance, a comprehensive post-optimization evaluation using precision, recall, F1-score, ROC, and PRC metrics is conducted, as described in Section 5.8.

### 5.3. Design Choice: Sequential vs. Simultaneous Optimization

In this study, feature selection and deep neural network hyperparameter optimization are performed sequentially rather than simultaneously. Feature selection is first conducted using biologically motivated filtering and differential gene expression analysis to ensure biological relevance and reduce dimensionality. Subsequently, AHALA is applied to optimize the hyperparameters of the deep neural network using the selected miRNA features.

This sequential strategy was intentionally adopted to (i) preserve biological interpretability of selected features, (ii) reduce the complexity of the search space, and (iii) improve optimization stability given the limited sample size. Simultaneous optimization of features and hyperparameters would significantly expand the solution space and increase the risk of overfitting, particularly in high-dimensional omics data.

Deep Neural Network Training: The differentially expressed genes identified through DGE analysis are used as input features for the deep neural network. AHALA optimizes the network’s parameters to maximize classification accuracy. The training process leverages the selected features to focus on the most informative and biologically relevant genes, resulting in a more accurate and efficient classification of breast cancer subtypes.

Biomarker Interpretation: To interpret the results of the classification task, we extract the most important biomarkers contributing to accurate predictions. This step enables us to identify key genes or miRNAs that serve as critical indicators for specific breast cancer subtypes. By identifying these biomarkers, we gain insights into the molecular mechanisms underlying breast cancer, offering potential targets for therapeutic intervention.

Data and Dataset: The study utilizes a publicly available dataset curated from The Cancer Genome Atlas (TCGA), which includes miRNA expression profiles and corresponding clinical information. The data Supplementary incorporates next-generation sequencing (NGS)-derived miRNA expression values, providing a rich resource for identifying miRNA biomarkers associated with the four primary molecular subtypes of breast cancer: Luminal A, Luminal B, HER2-enriched, and Basal-like [14]. Our methodology integrates data pre-processing, feature selection through DGE, and the novel AHALA optimization algorithm to train a deep neural network for accurate breast cancer subtype classification. This approach not only improves the efficiency of the classification process but also highlights key biomarkers for potential therapeutic targets.

### 5.4. Experimental Setup

This research used a miRNA expression dataset from breast cancer samples to train and test a neural network model optimized with a hybrid Hill Climbing and Adaptive Levy Algorithm (AHALA). The dataset, known as the miRNA-Combine-Dataset, was obtained from a combination of public breast cancer miRNA expression data, including TCGA-BRCA samples from the Cancer Genome Atlas Breast Invasive Carcinoma (TCGA-BRCA) repository. The experimental setup and dataset characteristics are summarized in Table 4.

#### 5.4.1. Dataset Description

The first dataset contains 231 samples, and each sample is defined by the expression levels of 588 miRNA genes [14]. The samples are divided into five classes according to breast cancer subtypes and a normal control: Luminal A (86 samples), Luminal B (39 samples), HER2-Enriched (24 samples), Basal-Like (41 samples), and Normal (41 samples). These classes constitute the significant molecular subtypes of breast cancer, where the Normal class is a control for differential expression analysis. The data was preprocessed to be compatible with machine learning tasks as follows.

#### 5.4.2. Differential Expression Analysis

In order to determine the differentially expressed genes (DEGs), we carried out differential expression analysis with the Limma package in R(2025.05.1 Build 513). We conducted the analysis on the miRNA dataset after genes were transposed into rows and samples into columns. The following steps were followed:

1. Preprocessing: Genes that had zero variance across samples were filtered out to exclude non-informative features. The expression values were then log2-transformed after a pseudocount of 1 was added to prevent undefined values (i.e., log2(expression + 1)).
2. Statistical Modeling: A linear model with Limma and a design matrix of the five sample classes (Luminal A, Luminal B, HER2-Enriched, Basal-Like, Normal) was utilized. Pairwise contrasts for all 10 comparisons among the five classes were specified (e.g., Basal-Like vs. Normal, Luminal A vs. Luminal B).
3. DEG Selection: Genes were chosen to be differentially expressed if they had an adjusted p-value (by the Benjamini-Hochberg method) of less than 0.05 and an absolute log2 fold change of more than 1. This narrowed down the number of genes from 588 to 208 DEGs, which were taken as input features for the neural network model.

The outcome of the differential expression analysis, i.e., the DEG list and statistics, is shown in Supplementary File S3.

### 5.5. Data Preprocessing for Machine Learning

The DEG expression data (231 samples, 208 genes) was standardized by StandardScaler from scikit-learn such that each feature has a mean of zero and a variance of one. Class labels were encoded by LabelEncoder, converting the five classes to integers (0 through 4). A stratified hold-out validation approach was used to split the data into training and test datasets such that 184 samples, 80% of the dataset, were used for the training set and 47 samples, 20%, for the test set. Both datasets, with relative proportions of Luminal A, Luminal B, HER2-Enriched, Basal-Like, and Normal samples, were ensured to maintain the class distribution following stratification.

### 5.6. Validation and Model

A fully connected three-layer feature extractor and a fully connected two-layer classifier constitute the neural network model, which has been implemented using PyTorch (version 2.4.1+cu121). AHALA framework, which blended local search via hill climbing with global search via adaptive Levy flights, optimized three hyperparameters: hidden size, learning rate, and batch size. For preventing overfitting, the network was trained using early stopping on validation loss with the Adam optimizer and cross-entropy loss. The test set was withheld for final evaluation only after model training and hyperparameter tuning, using a hold-out validation approach. This method is utilized to get an unbiased estimate of the model’s performance on unseen data. The performance of the model was evaluated against a set of metrics that involved accuracy, precision, recall, F1-score (weighted averages), and area under the receiver operating characteristic curve (AUC) for multi-class classification. Table 4 summarizes the dataset and experimental setup

The dataset is provided as Supplementary File S1, differentially expressed genes (DEGs) as Supplementary File S2, and the implementation code is available in Supplementary File S4 to facilitate reproducibility.

### 5.7. Differential Gene Expression Analysis Results

Differential gene expression (DGE) analysis was carried out to identify miRNA genes highly correlated with the five categories of samples in the miRNA-Combine-Dataset: Luminal A (86 samples), Luminal B (39 samples), HER2-Enriched (24 samples), Basal-Like (41 samples), and Normal (41 samples). We used the Limma package in R to carry out pairwise comparisons for all pairs of classes, producing 10 contrasts (e.g., Basal-Like versus Normal, and Luminal A versus Luminal B). Genes were considered differentially expressed if they met the criteria of adjusted *p*-value < 0.05 (Benjamini-Hochberg procedure) and absolute log2 fold change (|log2FC|) > 1. This narrowed down the starting 588 miRNA genes to 208 differentially expressed genes (DEGs), which were used as features for the neural network model.

**Figure 8:**
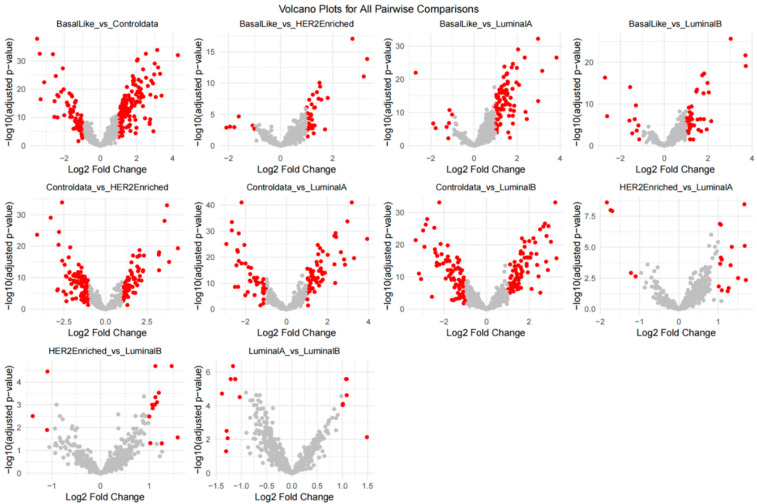
Volcano plots illustrating differentially expressed genes (DEGs) in breast cancer subtypes compared to normal controls, with red dots indicating significant DEGs (|log2FC| > 1, adjusted *p*-value < 0.05), highlighting molecular heterogeneity across Basal-Like, Luminal A, Luminal B, and HER2-Enriched subtypes.

The DGE analysis revealed certain miRNA expression patterns in the breast cancer subtypes and the Normal control group. Volcano plots, showing log2 fold change against the negative log10 of adjusted p-values for every contrast (see Figure 8, volcano plots). Volcano plots identify important DEGs with red dots signifying genes with the significance cut-off (|log2FC| > 1, adjusted *p*-value < 0.05). High-profile contrasts like Basal-Like vs. Normal and Luminal A vs. Luminal B had more significant DEGs, which were indicative of molecular heterogeneity between these subtypes. For example, the Basal-Like versus Normal phenotype comparison revealed miRNAs that were highly upregulated, implicating them in promoting aggressive tumor characteristics.

**Figure 9:**
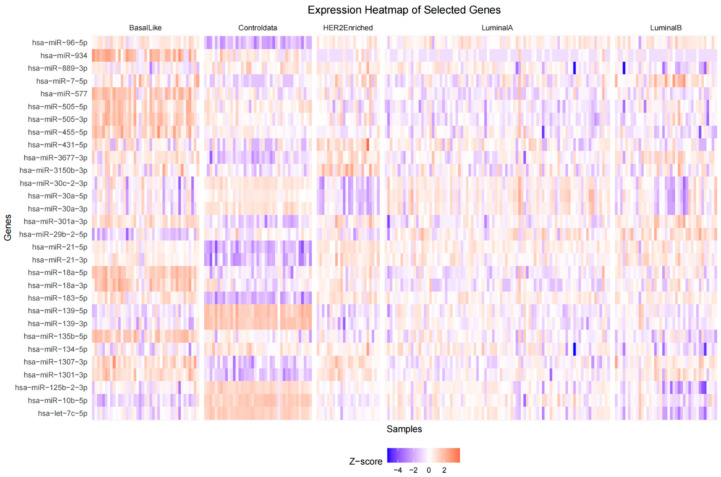
Heatmap of z-scored expression values for selected differentially expressed genes (DEGs) across breast cancer subtypes (Basal-Like, HER2-Enriched, Luminal A, Luminal B) and normal controls, showcasing distinct miRNA expression patterns.

**Figure 10:**
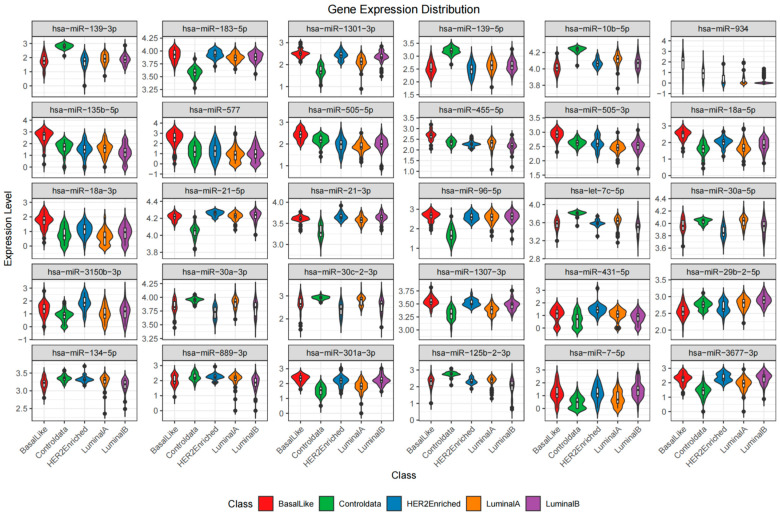
Violin plots depicting the expression distribution of selected differentially expressed genes (DEGs) across breast cancer subtypes (Basal-Like, HER2-Enriched, Luminal A, Luminal B) and normal controls.

The expression patterns of the most significant differentially expressed genes (DEGs) were depicted in a heatmap and violin plots. The heatmap (Figure 9) presents the z-scored expression values of 26 chosen DEGs across all samples, separated by class (Basal-Like, Controldata [Normal], HER2-Enriched, Luminal A, Luminal B). Replicative DEGs with distinctive expression patterns were hsa-miR-21-5p, hsa-miR-139-5p, hsa-miR-183-5p, and hsa-miR-30a-5p. For example, as compared to normal, hsa-miR-21-5p showed significant expression in HER2-Enriched and Basal-Like samples, in line with its known function to facilitate oncogenesis in breast cancer. Luminal A and Luminal B, respectively, had downregulated hsa-miR-139-5p, implying a tumor-suppressive function in the latter subtypes. Further details regarding the distribution of expression levels of these DEGs across the five subtypes are available in the violin plots (Figure 10). These plots show considerable expression variation within each subtype, particularly for hsa-miR-183-5p and hsa-miR-135b-5p in Basal-Like samples, thereby highlighting the existence of intra-subtype heterogeneity. In addition, the violin plots support the differential expression patterns observed in the heatmap, with several microRNAs showing statistically significant differences in median expression when cancer subtypes are compared to Normal samples.

The DEGs featured herein uncover some of the molecular mechanisms underlying the breast cancer subtypes. For example, as might be expected given its function, hsa-miR-21-5p, a well-characterized oncomiR in proliferation and invasion, was notably increased in Basal-Like and HER2-Enriched subtypes. Downregulated in all Luminal subtypes, Hsa-miR-139-5p is a tumor suppressor and can be utilized as a biomarker to differentiate between aggressive and Luminal A and B subtypes. These results reflect just how functional DGE analysis can be for determining miRNA signatures specific to a given subtype that can guide treatment strategies specific to that subtype.

### 5.8. Performance Evaluation of AHALA-miRNA for Breast Cancer Subtyping

In this subsection, we evaluate the performance of the proposed AHALA-miRNA framework for multi-class breast cancer subtyping using a comprehensive set of evaluation metrics, including Accuracy, Precision, Recall (True Positive Rate, TPR), False Positive Rate (FPR), F1-score, and the Area Under the Curve (AUC) derived from Receiver Operating Characteristic (ROC) and Precision–Recall Curves (PRCs). Since the problem involves five classes (Luminal A, Luminal B, HER2-Enriched, Basal-Like, and Normal), all metrics are computed following standard multi-class extensions.

For class-specific metrics, a one-vs-rest (OvR) strategy is employed, where each subtype is treated as the positive class while all remaining subtypes are grouped as the negative class. This allows the computation of binary metrics for each subtype independently.

True Positive Rate (TPR/Recall): For each class $c_{i}$, TPR is computed as:

(22) $${TPR}_{c}=\frac{{TP}_{c}}{{TP}_{c}+{FN}_{c}}$$

where $TP_{c}$ and $FN_{c}$ denote the true positives and false negatives for the class c, respectively. This metric reflects the model’s ability to correctly identify samples belonging to a given breast cancer subtype.

False Positive Rate (FPR): FPR for each class is calculated as:

(23) $${FPR}_{c}=\frac{{FP}_{c}}{{FP}_{c}+{TN}_{c}}$$

where $FP_{c}$ and $TN_{c}$ represent false positives and true negatives for the class c. A lower FPR indicates reduced misclassification of other subtypes as the target subtype.

Precision: Precision for each class is defined as:

(24) $${Precision}_{c}=\frac{{TP}_{c}}{{TP}_{c}+{FP}_{c}}$$

It measures the reliability of positive predictions for each subtype, indicating how often predicted subtype labels are correct.

F1-Score: The F1-score is computed as the harmonic mean of Precision and Recall for each class:

(25) $${F1}_{c}=\frac{2\times {Precision}_{c}\times {Recall}_{c}}{{Precision}_{c}+{Recall}_{c}}$$

This metric provides a balanced assessment, particularly important in the presence of class imbalance across breast cancer subtypes.

Accuracy: Overall classification accuracy is calculated as the proportion of correctly classified samples across all classes:

(26) $${Accuracy}_{c}=\frac{\sum_{c}{TP}_{c}}{N}$$

where N is the total number of samples. While accuracy offers a global performance indicator, it is interpreted alongside class-wise metrics to avoid bias due to class imbalance.

ROC and PRC Curves (Multi-class): ROC and PRC curves are generated using the one-vs-rest strategy for each class based on predicted class probabilities. The Area Under the Curve (AUC) is computed for each subtype independently and then aggregated using macro-averaging, which assigns equal weight to each class. This approach ensures fair evaluation across both majority and minority subtypes.

By employing these multi-class evaluation strategies, we provide a robust and statistically sound assessment of the AHALA-miRNA framework, demonstrating its effectiveness in distinguishing breast cancer subtypes while accounting for class imbalance and clinical relevance.

#### 5.8.1. Comparative Analysis and Discussion of Results

To robustly evaluate the performance of the Adaptive Hill Climbing Artificial Lemming Algorithm (AHALA-miRNA) for hyperparameter optimization in miRNA-based breast cancer subtyping, we first compared it against six established optimization algorithms, and second, compared it against two established gene selection approaches.

##### Comparative Analysis with Baseline Optimization Methods

**Figure 11:**
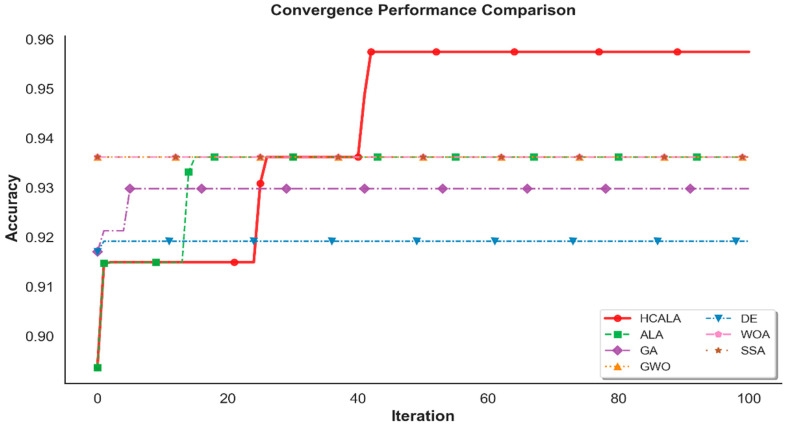
Convergence Curves of AHALA-miRNA and Baseline Optimization Methods.

In this sub-section, we compare the performance of the Adaptive Hill Climbing Artificial Lemming Algorithm (AHALA-miRNA) in hyperparameter optimization for miRNA-based breast cancer subtyping with that of six traditional optimization algorithms: Differential Evolution (DE-miRNA) [36], Artificial Lemming Algorithm (ALA-miRNA), Genetic Algorithm (GA-miRNA) [37], Grey Wolf Optimizer (GWO-miRNA) [38], Whale Optimization Algorithm (WOA-miRNA) [39], and Salp Swarm Algorithm (SSA-miRNA) [40]. These methods were selected based on their overall popularity in neural network hyperparameter optimization and readiness for use against the TCGA miRNA expression data (231 samples, 208 miRNAs after low-variance filtering and differential gene expression analysis). Comparison considers best accuracy, worst accuracy, best AUC, worst AUC, average accuracy, average AUC, runtime, and statistical significance in 10 runs, population size 30 and 100 iterations, run on a high-performance computing environment (AMD EPYC 7552, 48 cores, 1TiB RAM, Advanced Micro Devices, Inc., Santa Clara, CA, USA) with Python 3.9, PyTorch 1.10. The performance data are compiled in Table 5, and the convergence curves of the mean best-so-far accuracy over 100 iterations for each approach are shown in Figure 11.

Table 5 shows evidence of support for the dominance of AHALA-miRNA over six benchmarked baseline methods by its best performance in hyperparameter tuning in miRNA-based breast cancer subtyping. From observing its same best and worst accuracies and zero standard deviation, AHALA-miRNA produces the highest average accuracy of 0.9574 ± 0.0000, which is better compared to DE-miRNA’s best accuracy of 0.9574 but with less variability. Its best AUC (0.9733 ± 0.0000) is somewhat less than some baselines (e.g., GWO-miRNA, WOA-miRNA, SSA-miRNA at 0.9895), but its worst AUC (0.9733) is competitive, suggesting stable classification performance over runs. AHALA-miRNA’s runtime (676.19 ± 45.81 s, ~11.3 min) is among the lowest, being exceeded only by SSA-miRNA (765.96 s) and far lower than DE-miRNA (3233 s) and WOA-miRNA (4135 s).

Although DE-miRNA is a good overall baseline (average accuracy = 0.9191 ± 0.0185, AUC = 0.9784 ± 0.0063), it is not as effective for iterative optimization since it has an extremely long runtime (3233 s) and variability (std = 0.0185). ALA-miRNA, a component of AHALA, demonstrates the efficiency advantages of employing Hill Climbing’s local search by achieving good accuracy (0.9362 ± 0.0000) at over twice the runtime (1511 s). However, GA-miRNA attains competitive accuracy (0.9298 ± 0.0136), with instability and increased runtime (1178.48 s).

WOA-miRNA, SSA-miRNA, and GWO-miRNA have equal mean accuracy (0.9362 ± 0.0000) and AUC (0.9895 ± 0.0000), but their runtimes (829.53 s, 4135 s, and 765.96 s, respectively) are quite different, with WOA-miRNA being the slowest. The zero standard deviation of ALA-miRNA, GWO-miRNA, WOA-miRNA, SSA-miRNA, and AHALA-miRNA points to stable performance, likely owing to deterministic initialization based on fixed seeds (42 + run index), which may, however, limit exploration diversity in some cases.

Wilcoxon signed-rank tests (Table 5) also confirm AHALA-miRNA’s statistical superiority to all baselines, with p-values 0.0066 (DE-miRNA), 0.0062 (GA-miRNA), and 0.0020 (ALA-miRNA, GWO-miRNA, WOA-miRNA, SSA-miRNA), all below the 0.05 margin. These confirm again that AHALA-miRNA consistently outperforms the baselines across all 10 runs in terms of accuracy, confirming the effectiveness of its hybrid design by leveraging ALA’s global search and Hill Climbing’s local search efficiency.

Figure 11 illustrates AHALA-miRNA’s quicker convergence to 0.95 accuracy at iteration 50, compared to DE-miRNA (0.92 at iteration 100), ALA-miRNA (0.936 at iteration 100), and others. This is due to AHALA’s intermittent hill-climbing improvements (every 10 iterations), which enhance exploitation without sacrificing the global search of ALA. The moderate size of the TCGA dataset (231 samples, 208 miRNAs after preprocessing) is well leveraged by AHALA’s performance, as its running time is comparable to SSA-miRNA and GWO-miRNA but with better accuracy. These findings point to AHALA-miRNA’s effectiveness and power for miRNA-based breast cancer subtyping, particularly for moderately sized datasets like TCGA.

##### Comparative Analysis with miRNA Gene Selection Methods

The performance of the proposed AHALA-miRNA method for breast cancer subtyping is compared against two other approaches—CFS_SVM and CFS_RF [41], as shown in Table 6. The results for each subtype are evaluated across multiple performance metrics, including True Positive Rate (TPR), False Positive Rate (FPR), Precision, Recall, F-Measure, and Accuracy. Below, we provide a detailed discussion of the results.

Luminal A (LumA): The AHALA-miRNA method demonstrates a perfect True Positive Rate (1.00), indicating it successfully identifies all Luminal A samples, outperforming both CFS_SVM (0.78) and CFS_RF (0.89). The False Positive Rate (0.06) is also significantly lower than CFS_RF (0.16), suggesting that AHALA-miRNA generates fewer false positives. In terms of Precision, AHALA-miRNA achieves a high score of 0.90, which is superior to both CFS_SVM (0.82) and CFS_RF (0.76). The overall F-Measure for AHALA-miRNA is 0.95, demonstrating its superior balance between Precision and Recall, resulting in a notable improvement in classification accuracy (0.9362) compared to CFS_SVM (0.7660) and CFS_RF (0.8058).

Luminal B (LumB): The AHALA-miRNA model performs exceptionally well for the Luminal B subtype, with a True Positive Rate of 0.88, significantly outperforming CFS_SVM (0.50) and CFS_RF (0.40). Its False Positive Rate (0.02) is also impressively low compared to the other models. Precision and Recall are both 0.88 for AHALA-miRNA, showcasing a well-rounded performance, while CFS_SVM and CFS_RF display lower values in these areas. The F-Measure for AHALA-miRNA (0.88) underscores its improved classification balance over the comparative methods, highlighting the robustness of the proposed approach for this subtype.

HER2-Enriched (H2): AHALA-miRNA achieves strong results for the HER2-Enriched subtype, with a True Positive Rate of 0.80, surpassing both CFS_SVM (0.67) and CFS_RF (0.67). Moreover, AHALA-miRNA’s False Positive Rate (0.00) indicates perfect specificity, unlike CFS_RF (0.02) and CFS_SVM (0.07). Precision (1.00) and the F-Measure (0.89) are also markedly higher for AHALA-miRNA, reflecting its enhanced ability to correctly classify HER2-Enriched samples with minimal error. These results are indicative of the model’s improved reliability and precision for this particular subtype.

Basal-Like (BL): AHALA-miRNA demonstrates high performance for Basal-Like cancer with a True Positive Rate of 0.88, closely matching CFS_SVM (0.83) and CFS_RF (1.00). Notably, AHALA-miRNA achieves a False Positive Rate of 0.00, outperforming CFS_RF (0.02). Precision (1.00) and F-Measure (0.94) for AHALA-miRNA are also higher than those of CFS_SVM and CFS_RF, reflecting a better overall performance in accurately identifying Basal-Like samples while minimizing false positives.

Normal Samples: For normal samples, AHALA-miRNA shows perfect performance with a True Positive Rate, Precision, and F-Measure of 1.00, consistent with CFS_RF but exceeding CFS_SVM’s performance. Additionally, the False Positive Rate for AHALA-miRNA is 0.00, demonstrating perfect specificity, making it the most reliable model for classifying normal samples.

Across all subtypes, the proposed AHALA-miRNA model consistently outperforms the CFS_SVM and CFS_RF methods, particularly in terms of True Positive Rate, Precision, and F-Measure. The significant improvements in the False Positive Rate further underline the effectiveness of AHALA-miRNA in reducing misclassification rates. These results confirm that AHALA-miRNA not only enhances the classification accuracy for breast cancer subtypes but also maintains a strong balance between sensitivity and specificity, making it a robust approach for breast cancer subtyping and classification.

### 5.9. Computational Efficiency and Optimization Effectiveness

To compare the computational complexity and optimization capability of the Adaptive Hill Climbing Artificial Lemming Algorithm (AHALA) and its constituents—Hill Climbing and the Artificial Lemming Algorithm (ALA)—experiments were performed on the TCGA miRNA expression dataset (231 samples, 208 miRNAs after excluding low-variance features and differential gene expression analysis). The goal was to compare AHALA as a hyperparameter tuning method (hidden size, learning rate, batch size) for a neural network for breast cancer subtyping based on miRNA, amidst skepticism regarding the efficacy of Hill Climbing for medical data. We compared runtime, plotted convergence curves, and conducted statistical comparisons between the three methods, presenting a balanced view of their performance.

Experiments were run on a high-performance computing facility (AMD EPYC 7552, 48 cores, 1TiB RAM) using Python 3.9, PyTorch 1.10, and opfunu for optimization experiments. Every algorithm was executed 10 times with varied random seeds (42 + run index) for consistency, optimizing a 3D search space (hidden size: [64,512], learning rate: [0.0001, 0.01], batch size: [16,128]) for 100 iterations for ALA and AHALA, and Hill Climbing. Execution time was measured using the time module, convergence plots were generated using Matplotlib, and Wilcoxon signed-rank tests were conducted using SciPy for accuracy distribution comparison. The computer code utilized to derive these findings can be found at https://github.com/guino001/HCALA_miRNA (accessed on 3 February 2026). Table 5 summarizes accuracy and running time results, Figure 8 shows the convergence curves, and Table 7 summarizes the results of statistical tests, with all of these being averages of 10 runs.

**Figure 12:**
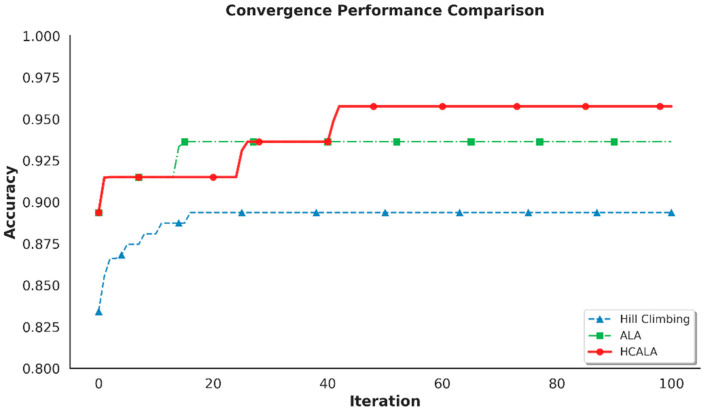
Convergence Curves of AHALA and Its Components.

Figure 12 graphs average best-so-far accuracy versus iteration for 10 runs of Hill Climbing, ALA, and AHALA (100 iterations). Hill Climbing converges rapidly but levels off at a lower accuracy of 0.8894. ALA improves steadily, reaching 0.9362 at iteration 100. AHALA converges more rapidly than ALA, reaching 0.9574 at iteration 50, thanks to periodic Hill Climbing improvement every 10 iterations.

The outcome shows that for TCGA miRNA data, AHALA achieves a more appropriate balance between optimization quality and computational speed. As the fastest one with the running time (mean = 27.69 s), Hill Climbing is a simple local search algorithm with adaptive step-size decrease (step_size = 0.01) and comparatively few random perturbations. However, as represented by the steep flattening of its convergence curve (Figure 12), its sensitivity to local optima negatively affects its accuracy (mean = 0.8894, std = 0.0519). As global search dominates in complex medical datasets, Hill Climbing does not work effectively as a standalone method. ALA, via its bio-inspired mechanisms (i.e., migration, burrowing), finds greater accuracy (mean = 0.9362) at the expense of significantly longer runtimes (mean = 1511.95 s, ~25.2 min). Its convergence plot is one of steady improvement, indicating good global search, but at this level of computational expense, its applicability is poor in practice for iterative hyperparameter tuning. ALA’s standard deviation of zero for accuracy (std = 0.0000) is a sign of consistent behavior across runs, possibly due to its deterministic initialization with fixed seeds, but potentially also a sign of a lack of exploration diversity for some runs.

AHALA integrates the advantages of both parts, having the best accuracy (mean = 0.9574) with a moderate running time (mean = 676.19 s, ~11.3 min). Its convergence graph (Figure 12) demonstrates quicker convergence than ALA at ~0.95 by iteration 50 due to periodic Hill Climbing updates every 10 iterations that enhance local exploitation. AHALA’s zero standard deviation of accuracy (std = 0.0000) echoes the reliability of ALA, highlighting the effective combination of global and local search processes. The reduction in runtime relative to ALA (56% fewer) speaks to the efficiency realized by Hill Climbing’s low-overhead perturbations, and makes AHALA particularly suitable for the TCGA dataset’s relatively small size (231 samples, 208 miRNAs). Wilcoxon signed-rank tests (Table 8) also confirm statistically significant better performance of AHALA over Hill Climbing and ALA (*p* = 0.0020 for both), with a statistic of 0.00 meaning that AHALA performed better than its components in every single run. This verifies the effectiveness of the hybrid approach, where the local search of Hill Climbing counteracts ALA’s slow convergence, and the global search of ALA counteracts Hill Climbing’s local optima traps. The p-value of 0.0020 (lower than the 0.05 level) verifies the significance of AHALA’s improved performance.

### 5.10. Discussion of Classification and Gene Importance Results

**Figure 13:**
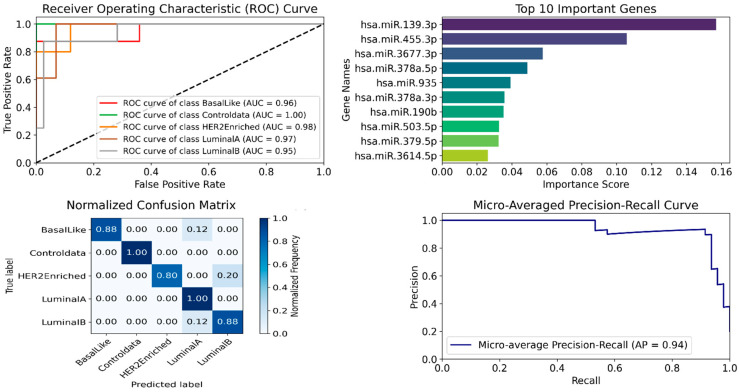
Receiver Operating Characteristic (ROC) Curve, Top 10 Importance Genes, Normalized Confusion Matrix, Micro-Averaged Precision-Recall Curve.

Figure 13 provides a comprehensive visualization of the performance of the proposed AHALA-miRNA method across multiple metrics and components, specifically targeting breast cancer subtypes.

Receiver Operating Characteristic (ROC) Curve: The ROC curves in the top left of Figure 13 highlight the AUC (Area Under Curve) values for each breast cancer subtype, as well as for the control group. The AHALA-miRNA method demonstrates excellent discriminative ability across all subtypes, with AUC values ranging from 0.95 for Luminal B to 1.00 for the control data. Notably, HER2-Enriched and Basal-Like subtypes also exhibit high AUC scores of 0.98 and 0.96, respectively. These AUC values illustrate the robustness of the model in distinguishing between cancer subtypes and the control group, confirming its high sensitivity and specificity.

Top 10 Important Genes: The top-right section of Figure 13 presents the ten most important miRNAs identified by the AHALA-miRNA method for breast cancer subtyping. The gene hsa.miR.139.3p exhibits the highest importance score, followed by hsa.miR.455.3p and hsa.miR.3677.3p. The importance of these genes suggests that they play a critical role in differentiating between the breast cancer subtypes, which could provide valuable insights for future research and potential therapeutic targets.

Normalized Confusion Matrix: The bottom-left plot shows the normalized confusion matrix, which visualizes the classification performance for each subtype. The matrix indicates a high degree of accuracy in predicting the control group and Luminal A subtype, with perfect classification (normalized value of 1.00). Similarly, for the Basal-Like and Luminal B subtypes, the model achieves strong classification, with diagonal values of 0.88, showcasing its reliability in these categories. Some misclassifications are observed between HER2-Enriched and Luminal A, as reflected by off-diagonal values, where 20% of HER2-Enriched samples are misclassified as Luminal A. However, overall performance remains high across subtypes.

Micro-Averaged Precision-Recall Curve: The micro-averaged precision-recall curve in the bottom-right corner of Figure 12 illustrates the model’s ability to balance precision and recall across all classes. The average precision (AP) score of 0.94 demonstrates the model’s excellent ability to retrieve relevant instances with minimal false positives, further affirming the model’s efficacy in handling imbalanced datasets. This curve complements the ROC curve analysis by providing a detailed view of precision and recall trade-offs, especially useful in evaluating model performance for individual subtypes.

Figure 13 showcases the high classification performance of the AHALA-miRNA model, with superior AUC values, a strong gene selection process identifying biologically relevant miRNAs, and balanced precision-recall curves. These results solidify the method’s potential for accurate breast cancer subtyping and biomarker identification.

### 5.11. Kaplan–Meier Survival Analysis and Expression Analysis of Selected miRNAs

**Figure 14:**
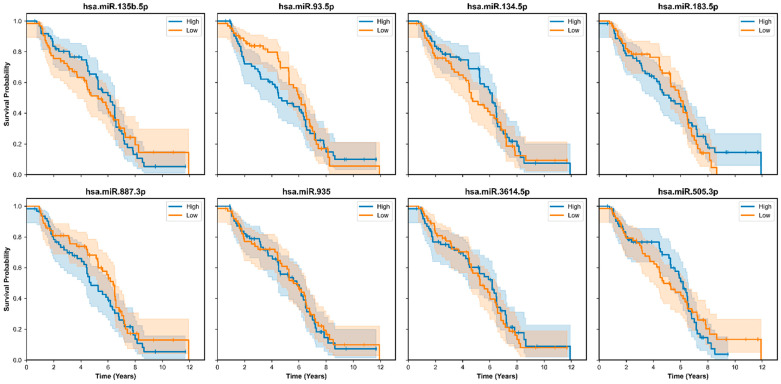
Survival Analysis (Kaplan–Meier) for Selected miRNA genes.

To examine the prognostic relevance of miRNA expression in our study group, we conducted a Kaplan–Meier survival analysis for the eight prominent miRNAs determined through the AHALA method. The miRNAs in question were hsa.miR.135b.5p, hsa.miR.93.5p, hsa.miR.134.5p, hsa.miR.183.5p, hsa.miR.887.3p, hsa.miR.935, hsa.miR.3614.5p, and hsa.miR.505.3p. Patients were classified into “High” and “Low” expression groups for each miRNA based on the median expression value. Survival time, quantified as days_to_death/days_to_last_followup and converted to years for ease of interpretation, was used as the time variable, and the Status column (1 = event/death, 0 = censored) was used to specify the event of interest. Kaplan–Meier survival curves were estimated to compare survival probabilities between high and low expression groups for all miRNAs, and statistical significance was determined using the log-rank test. The results are presented in a 2×4 grid of Kaplan–Meier plots, as shown in Figure 14.

The survival plots of both miRNAs are shown in Figure 14, with orange and blue indicating the low-expression and high-expression groups, respectively. The log-rank p-values in the subplots show statistical significance of the difference in survival between the two groups. It is interesting to notice hsa.miR.887.3p shows the greatest difference (*p* = 0.0763) and suggests the potential that the expression level could be associated with patient survival. Compared to the low expression group, the high expression group contains a declining survival probability over time, according to this miRNA’s survival plots. Similarly, hsa.miR.134.5p (*p* = 0.0837) and hsa.miR.93.5p (*p* = 0.0874) showed p-values approaching significance, indicating possible prognostic relevance. For comparison, miRNAs such as hsa.miR.505.3p (*p* = 0.9689) and hsa.miR.3614.5p (*p* = 0.9089) did not show any statistically significant difference in survival between the expression groups, since their survival curves were discovered to overlap significantly within the monitoring period.

Univariate Cox proportional hazards regression was performed for each individual miRNA to further evaluate the relationship between the miRNA level of expression and survival outcomes. The findings, as presented in Table 9, provide the Cox coefficient, hazard ratio (HR), 95% confidence interval (CI) of the HR, the unadjusted p-value, and p-value adjusted for false discovery rate (FDR) using the Benjamini-Hochberg procedure. While the results were not statistically significant (*p* = 0.0874, FDR = 0.233), Cox regression analysis indicated that hsa.miR.93.5p exhibited the highest significant hazard ratio (HR = 1.302, 95% CI: 0.962–1.761), indicating that its overexpression can be linked with a higher risk of mortality. Similarly, hsa.miR.887.3p also showed an HR of 1.273 (95% CI: 0.975–1.663, *p* = 0.0763, FDR = 0.233), in line with the Kaplan–Meier findings and indicating an unfavorable prognostic impact. In contrast, microRNAs such as hsa.miR.135b.5p (HR = 0.781, 95% CI: 0.573–1.066, *p* = 0.1196, FDR = 0.239) and hsa.miR.134.5p (HR = 0.801, 95% CI: 0.623–1.030, *p* = 0.0837, FDR = 0.233) presented hazard ratios below 1, suggesting a possible protective function; however, these observations did not achieve statistical significance following FDR adjustment. The rest of the miRNAs, including hsa.miR.183.5p, hsa.miR.935, hsa.miR.3614.5p, and hsa.miR.505.3p, exhibited HR close to 1 and high *p*-values (all > 0.6, FDR > 0.9), indicating a lack of association with survival.

The combination of Kaplan–Meier and Cox analysis indicates that hsa.miR.887.3p, hsa.miR.93.5p, and hsa.miR.134.5p may be of prognostic significance in this cohort and thus deserve further study in larger cohorts. Nevertheless, the failure to achieve statistical significance following false discovery rate (FDR) correction necessitates the interpretation of these findings with caution, given that the sample size may not provide sufficient power to identify small effect sizes. Validation of these results and insight into the biological functions of these miRNAs in cancer progression may be provided by future research using larger populations and more clinical covariates.

### 5.12. Pathway Enrichment Analysis of Selected miRNAs

To understand the biological meaning of their candidate target genes, the target microRNAs (miRNAs) were initially identified and then enriched for pathways. Pathway enrichment analysis was performed using clusterProfiler (v4.0) [42], an extensively validated R package for comparing biological themes. Target genes were first predicted by integrating results from several databases (TargetScan, miRanda, and DIANA-microT) using the miRNAtap algorithm [43], which calculates consensus targets using a geometric mean strategy (requires at least two prediction sources). Analysis included all of the top 20 selected miRNAs (hsa-mir-135b, hsa-mir-93, hsa-mir-134, hsa-mir-183, hsa-mir-887, hsa-mir-935, hsa-mir-3614, hsa-mir-505, hsa-mir-429, hsa-mir-190b, hsa-mir-99b, hsa-mir-130a, hsa-mir-501, hsa-mir-223, hsa-mir-21, hsa-mir-337, hsa-mir-19a, hsa-mir-27a, hsa-mir-452, and hsa-mir-10a) with human (Homo sapiens) gene annotations.

**Figure 15:**
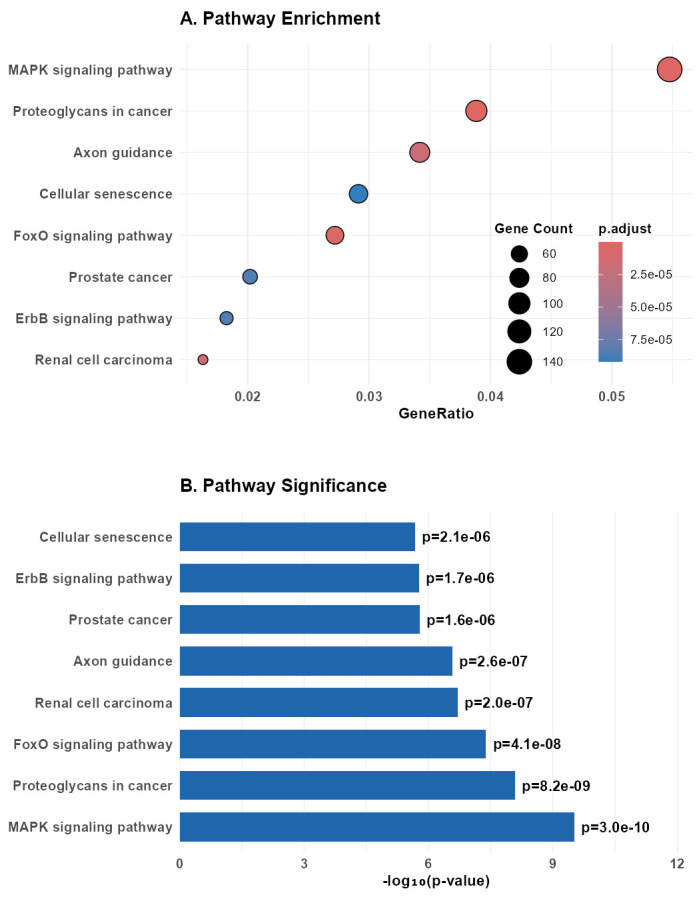
Pathway enrichment analysis of miRNA target genes (**A**) Dot plot showing significantly enriched KEGG pathways. Dot size represents gene count; color indicates adjusted *p*-value. (**B**) Bar plot ranking pathways by statistical significance (−log10(*p*-value)). Exact *p*-values are labeled. Data sourced from the KEGG pathway database (Kanehisa Laboratories), used with permission [44].

For pathway mapping, we employed KEGG enrichment [44] under the following conditions: Benjamini-Hochberg-corrected *p*-value threshold of 0.05, and false discovery rate (FDR) cutoff < 0.1. The analysis identified 12 significantly enriched pathways (*p* < 0.05), and the top hits are displayed in Figure 15. Figure 15A displays the pathways as a dot plot, where dot size is proportional to gene number (60–140 targets per pathway) and color gradient to statistical significance (adjusted *p*-values: 2.5 × 10^−5^ to 7.5 × 10^−5^).

Importantly, MAPK signaling pathway (adjusted *p* = 3.0 × 10^−10^), proteoglycans in cancer (adjusted *p* = 8.2 × 10^−9^), and renal cell carcinoma (adjusted *p* = 2.0 × 10^−7^) were the most significant, suggesting the role of these miRNAs in proliferation of tumor cells, extracellular matrix remodeling, and metastatic signaling—processes that are always involved in the development of breast cancer [45].

The consistency of such correlations is also shown in Figure 15B, in which the pathways are ordered by −log10(*p*-value). The dramatic significance of the MAPK pathway (*p* = 3.0 × 10^−10^) agrees with its established involvement in treatment resistance [46], whereas the enrichment in proteoglycans indicates a possible modulation of the tumor microenvironment. These in silico predictions, while requiring empirical verification (e.g., by miRNA mimic/inhibitor assays), offer a mechanistic foundation for the interpretation of the functional roles of miRNAs in specific subtypes of breast cancer.

All these findings suggest that the miRNAs described here can be candidate markers or therapeutic targets because they regulate pathways that are crucial to cancer progression.

### 5.13. Biological Relevance of Identified miRNAs in Breast Cancer Subtyping

The miRNAs identified by the proposed AHALA-miRNA framework demonstrate strong biological relevance, as many of them have been previously implicated in breast cancer initiation, progression, and subtype-specific molecular regulation. Importantly, these miRNAs were consistently selected by the optimization algorithm and further validated through pathway enrichment analysis (Section 5.12), reinforcing their functional significance.

Among the most prominent miRNAs, hsa-mir-21 is a well-established oncogenic miRNA known to promote tumor proliferation, invasion, and metastasis through the regulation of tumor suppressor genes and key signaling cascades. Its involvement in cancer-related pathways aligns with the significant enrichment of the MAPK signaling pathway, which plays a central role in breast cancer progression and therapeutic resistance [47,48].

The biomarkers hsa-mir-93 [49,50] and hsa-mir-135b [51,52] have been reported to regulate cell cycle progression, angiogenesis, and oncogenic signaling. These miRNAs are frequently associated with aggressive tumor behavior and are consistent with the enrichment of pathways related to cell proliferation and extracellular matrix remodeling, including proteoglycans in cancer.

In addition, hsa-mir-183 [53,54] and hsa-mir-130a [55] are involved in the regulation of epithelial–mesenchymal transition (EMT) and metastatic dissemination, further supporting their relevance to breast cancer subtype differentiation. The identification of these miRNAs suggests that the proposed framework captures regulatory elements linked to tumor invasiveness.

Furthermore, hsa-mir-99b [56] and hsa-mir-223 [57,58] have been associated with immune-related signaling and modulation of tumor–microenvironment interactions. Their enrichment in cancer-related pathways underscores the potential role of immune regulation in subtype-specific tumor behavior.

Overall, the convergence of algorithmic selection, pathway enrichment, and existing biological evidence indicates that the AHALA-miRNA framework effectively identifies miRNAs that are not only discriminative for classification but also mechanistically linked to key pathways involved in breast cancer development. This integrated computational–biological validation strengthens the potential utility of the identified miRNAs as biomarkers and therapeutic targets.

### 5.14. Evaluation on Independent Test Data

To ascertain the generalizability of our AHALA algorithm-optimized neural network model, we tested its performance on an independent test dataset, GSE58606, from the Gene Expression Omnibus (GEO) [59]. The GSE58606 dataset contains miRNA expression levels of 133 breast cancer and normal tissue samples measured on the Agilent Human miRNA Microarray (GPL14767). This dataset includes 133 tumor samples labeled LuminalA (31), LuminalB (33), HER2Enriched (27), BasalLike (31), and 11 Controldata (normal tissue) samples. These labels are directly mapped to the TCGA dataset’s classes for uniform evaluation.

Among the 208 miRNAs utilized in our TCGA-trained model, 181 were available in GSE58606, while 27 were absent owing to differences in microarray platforms. To make up for this, we imputed the absent miRNAs with label-specific median expression values from the TCGA training set. For every GSE58606 sample, the expression of a missing miRNA was imputed to the median expression of that miRNA across TCGA samples of the same subtype (for example, LuminalA medians for LuminalA samples), maintaining subtype-specific expression patterns. GSE58606 expression data was Z-score normalized first to normalize values across samples. The feature matrix was then aligned (133 samples × 208 miRNAs) and normalized by StandardScaler trained on the TCGA training data for compatibility with the training pipeline. Labels were encoded by LabelEncoder trained on the TCGA training labels, taking advantage of their shared naming convention.

The model was evaluated without retraining to see how it would perform on unseen data and yielded the following on GSE58606: Accuracy of 0.8297, Precision of 0.8425, Recall of 0.8297, F1 Score of 0.8261, AUC of 0.9497, and MCC of 0.7854. These metrics are compared with the TCGA test set results in Table 10.

The performance evaluation of our model using the GSE58606 dataset is satisfactory with an AUC of 0.9497 (Table 10), which indicates high discriminative capability across various subtypes of breast cancer and MCC of 0.7854 that validates its balanced classification capability, especially vital for multi-class problems that have class imbalances. However, these readings are lower than those achieved on the TCGA test dataset, where the model achieved a measure of accuracy equal to 0.9574 and AUC equal to 0.9682. The difference in performance can be due to several reasons. The TCGA dataset and GSE58606 were generated from different experimental platforms, including RNA-seq or other microarray technologies for TCGA, compared with the Agilent microarray for GSE58606, which generates miRNA quantification and expression profiles with differences that cause noise and distribution shifts, compromising the generalizability of the model. In addition, imputation of 27 missing miRNAs representing 13% of features in GSE58606 is from label-specific median expression values in TCGA, and while this is a biologically informed approach, it might not fully represent true expression patterns in GSE58606 samples and could introduce minimal biases, especially for highly subtype-variable miRNAs. The TCGA test set, derived from the identical dataset as in the training phase, has the advantage of homogeneous patient populations, preprocessing procedures, and experimental conditions, which are likely to enhance its performance. GSE58606, being an independent dataset with an independent patient cohort and a smaller test set of 133 samples in contrast to the total of 231 samples in TCGA, presents higher variability that destabilizes performance. Nevertheless, the GSE58606 result is competitive in accuracy at 0.8297 and AUC at 0.9497, with good generalizability to an independent cohort, and MCC at 0.7854, affirming balanced performance across subtypes, correcting for potential class imbalances. The results demonstrate the strength of the model and its applicability in clinical settings for the classification of breast cancer subtypes, but also point out directions for possible future improvement.

## 6. Conclusions

This study introduces the Adaptive Hill Climbing Artificial Lemming Algorithm (AHALA) as an effective tool for breast cancer subtype classification and deep neural network optimization. By integrating low-variance gene filtering, differential gene expression analysis, and AHALA-driven feature selection and model tuning, we achieved robust classification of Luminal A, Luminal B, HER2-enriched, and Basal-like subtypes using TCGA miRNA expression data. The identification of key miRNA biomarkers, including hsa-miR-190b, hsa-miR-429, and hsa-miR-935, underscores AHALA’s potential for biological insight and optimization, striking a balance between exploration and exploitation to avoid local optima. AHALA’s ability to refine high-dimensional search spaces and enhance model performance highlights its versatility beyond traditional optimization techniques. Furthermore, its application to publicly available datasets like TCGA ensures reproducibility and broad applicability in cancer research. These findings position AHALA as a valuable asset for precision medicine, offering a scalable approach to tackling complex biological datasets. Future efforts could refine AHALA for broader applications, explore the functional roles of identified biomarkers, and investigate its performance on other cancer types, advancing diagnostics and personalized treatment strategies.

## Figures and Tables

**Table 1:** Algorithm-Specific Parameter Configurations.

Algorithm	Parameters Specifications
RIME	Soft-rime(sr) = 5.0; popSize = 30;
QleSCA	learning rate in Q-learning (alpha) = 0.1; (gama) = 0.9;
EnhancedTWO	popSize = 30;
RWGWO	popSize = 30;
GBO	popSize = 30;
AHALA	max_iterations = 10, step_size = 0.01

**Table 2:** Comparison with some recent optimizers using CEC2021.

Algorithm	Criteria	f1	f2	f3	f4	f5	f6	f7	f8	f9	f10
RW_GWO	Avg	2.3973 × 10^4^	2.0381 × 10^3^	8.3335 × 10^2^	1.9043 × 10^3^	8.3447 × 10^4^	6.4952 × 10^3^	1.0310 × 10^5^	2.3015 × 10^3^	2.6460 × 10^3^	3.2061 × 10^3^
	Std	1.7419 × 10^4^	3.9514 × 10^2^	2.6949 × 10^1^	1.8315 × 10^0^	3.8213 × 10^4^	3.7659 × 10^3^	1.2606 × 10^5^	4.5530 × 10^0^	6.4145 × 10^1^	7.0104 × 10^1^
	Med	2.0246 × 10^4^	1.9874 × 10^3^	8.3556 × 10^2^	1.9039 × 10^3^	7.4007 × 10^4^	6.6262 × 10^3^	6.1869 × 10^4^	2.3004 × 10^3^	2.6157 × 10^3^	3.1804 × 10^3^
RIME	Avg	1.4415 × 10^4^	**1.7448 × 10^3^**	7.9400 × 10^2^	1.9035 × 10^3^	8.2134 × 10^4^	2.0760 × 10^3^	5.3691 × 10^4^	2.3064 × 10^3^	2.6159 × 10^3^	3.2044 × 10^3^
	Std	7.4648 × 10^3^	2.4758 × 10^2^	2.2325 × 10^1^	1.0194 × 10^0^	4.2140 × 10^4^	3.7309 × 10^2^	6.1219 × 10^4^	1.0655 × 10^1^	3.5137 × 10^1^	9.0281 × 10^1^
	Med	1.1871 × 10^4^	1.7205 × 10^3^	7.9626 × 10^2^	1.9034 × 10^3^	7.3490 × 10^4^	1.9712 × 10^3^	3.8207 × 10^4^	2.3002 × 10^3^	2.6068 × 10^3^	3.1684 × 10^3^
QleSCA	Avg	1.8437 × 10^10^	4.0321 × 10^3^	3.9299 × 10^5^	1.7036 × 10^5^	8.0697 × 10^6^	1.7864 × 10^5^	5.0905 × 10^7^	2.5071 × 10^3^	1.5392 × 10^4^	6.1822 × 10^3^
	Std	5.8113 × 10^9^	4.2980 × 10^2^	1.0597 × 10^5^	2.1006 × 10^5^	7.7253 × 10^6^	5.8032 × 10^5^	6.0325 × 10^7^	8.1714 × 10^1^	4.0262 × 10^3^	9.9614 × 10^2^
	Med	1.8202 × 10^10^	4.0390 × 10^3^	3.6952 × 10^5^	9.5951 × 10^4^	5.7577 × 10^6^	4.2676 × 10^4^	2.9348 × 10^7^	2.4998 × 10^3^	1.4967 × 10^4^	6.2415 × 10^3^
GBO	Avg	1.5320 × 10^5^	2.2472 × 10^3^	9.7244 × 10^2^	1.9101 × 10^3^	7.1869 × 10^4^	4.8373 × 10^3^	8.7908 × 10^4^	2.3301 × 10^3^	2.6310 × 10^3^	3.2296 × 10^3^
	Std	1.6359 × 10^5^	3.9832 × 10^2^	8.0081 × 10^1^	5.4593 × 10^0^	4.3277 × 10^4^	2.8883 × 10^3^	7.2769 × 10^4^	3.9216 × 10^0^	2.6267 × 10^1^	6.6838 × 10^1^
	Med	1.0768 × 10^5^	2.2097 × 10^3^	9.5693 × 10^2^	1.9091 × 10^3^	6.0133 × 10^4^	3.9532 × 10^3^	6.9424 × 10^4^	2.3298 × 10^3^	2.6303 × 10^3^	3.2402 × 10^3^
Enhanced TWO	Avg	2.0776 × 10^4^	2.5816 × 10^3^	8.3379 × 10^2^	1.9060 × 10^3^	9.4822 × 10^4^	4.6326 × 10^3^	8.2360 × 10^4^	**2.3002 × 10^3^**	2.6073 × 10^3^	3.1734 × 10^3^
	Std	3.3575 × 10^3^	4.7955 × 10^2^	1.1357 × 10^1^	1.6254 × 10^0^	6.4626 × 10^4^	3.0852 × 10^3^	1.0033 × 10^5^	1.7224 × 10^−2^	1.8807 × 10^1^	5.7439 × 10^1^
	Med	2.0609 × 10^4^	2.6276 × 10^3^	8.3624 × 10^2^	1.9057 × 10^3^	7.9493 × 10^4^	3.3277 × 10^3^	3.8187 × 10^4^	2.3002 × 10^3^	2.6107 × 10^3^	3.1447 × 10^3^
ALA	Avg	1.4208 × 10^3^	2.7820 × 10^3^	**7.7111 × 10^2^**	1.9034 × 10^3^	3.9413 × 10^3^	1.8578 × 10^3^	4.2559 × 10^3^	2.3073 × 10^3^	2.6106 × 10^3^	**3.1677 × 10^3^**
	Std	1.4879 × 10^3^	5.0301 × 10^2^	1.5278 × 10^1^	1.1148 × 10^0^	1.7114 × 10^3^	2.7483 × 10^2^	3.4013 × 10^3^	1.1581 × 10^1^	4.0326 × 10^1^	7.1380 × 10^1^
	Med	9.8627 × 10^2^	2.8013 × 10^3^	7.7048 × 10^2^	1.9035 × 10^3^	3.5381 × 10^3^	1.7285 × 10^3^	3.0429 × 10^3^	2.3000 × 10^3^	2.6000 × 10^3^	3.1425 × 10^3^
Proposed AHALA	Avg	**1.0280 × 10^2^**	2.1750 × 10^3^	7.8842 × 10^2^	**1.9037 × 10^3^**	**3.6358 × 10^3^**	**1.6169 × 10^3^**	**2.7639 × 10^3^**	2.3031 × 10^3^	**2.6070 × 10^3^**	3.1766 × 10^3^
	Std	4.1798 × 10^0^	3.7175 × 10^2^	2.7046 × 10^1^	1.5431 × 10^0^	2.9065 × 10^3^	2.7704 × 10^1^	1.0371 × 10^3^	8.8323 × 10^0^	3.8255 × 10^1^	4.9119 × 10^1^
	Med	1.0089 × 10^2^	2.2016 × 10^3^	7.8563 × 10^2^	1.9035 × 10^3^	2.8295 × 10^3^	1.6050 × 10^3^	2.4687 × 10^3^	2.3000 × 10^3^	2.6000 × 10^3^	3.1572 × 10^3^

**Table 3:** Wilcoxon signed-rank test (*p*-value) results of AHALA and other algorithms on CEC2021.

Function	Our Proposed AHALA Vs.					
	ALA	Enhanced TWO	GBO	QleSCA	RIME	RWGWO
f1	1.86 × 10^−9^	1.86 × 10^−9^	1.86 × 10^−9^	1.86 × 10^−9^	1.86 × 10^−9^	1.86 × 10^−9^
f2	8.86 × 10^−5^	0.00202	0.626346	1.86 × 10^−9^	9.90 × 10^−5^	0.09195
f3	0.01546	1.86 × 10^−9^	1.86 × 10^−9^	1.86 × 10^−9^	0.489846	2.08 × 10^−5^
f4	0.427955	5.14 × 10^−6^	2.05 × 10^−7^	1.86 × 10^−9^	0.776569	0.076721
f5	0.02085	1.86 × 10^−9^	1.86 × 10^−9^	1.86 × 10^−9^	1.86 × 10^−9^	1.86 × 10^−9^
f6	0.000189	1.86 × 10^−9^	1.86 × 10^−9^	1.86 × 10^−9^	1.86 × 10^−9^	1.86 × 10^−9^
f7	0.004665	1.86 × 10^−9^	1.86 × 10^−9^	1.86 × 10^−9^	1.86 × 10^−9^	1.86 × 10^−9^
f8	0.100421	0.013663	3.73 × 10^−9^	1.86 × 10^−9^	0.005013	0.012834
f9	1	0.000153	0.000137	1.86 × 10^−9^	3.79 × 10^−6^	3.79 × 10^−6^

**Table 4:** The dataset and experimental setup.

Attribute	Description
Dataset Name and Source	miRNA Dataset, [14]
Total Samples	231
Classes (Sample Counts)	Luminal A (86), Luminal B (39), HER2-Enriched (24), Basal-Like (41), Normal (41)
Original Genes	588
Genes after DEG Analysis	208
DEG Method	Linear modeling with limma (adjusted *p*-value < 0.05)
Data Split	80% training (184 samples), 20% testing (47 samples), stratified
Hyperparameter Optimization	AHALA (hidden size: [64,512], learning rate: [0.0001, 0.01], batch size: [16,128])

**Table 5:** Performance Comparison of AHALA-miRNA and Baseline Optimization Methods.

Method	Best Accuracy	Worst Accuracy	Best AUC	Worst AUC	Accuracy Avg. ± Std	AUC Avg. ± Std	Runtime (s) Avg. ± Std	Wilcoxon Signed-Rank Test Results (vs. AHALA)
DE-miRNA	0.9574	0.8936	0.9895	0.9686	0.9191 ± 0.0185	0.9784 ± 0.0063	3233 ± 1515.22	0.0066
ALA-miRNA	0.9362	0.9362	0.9863	0.9863	0.9362 ± 0.0000	0.9863 ± 0.0000	1511 ± 98.39	0.0020
GA-miRNA	0.9412	0.9149	0.9895	0.9722	0.9298 ± 0.0136	0.9799 ± 0.0051	1178.48 ± 414.77	0.0062
GWO-miRNA	0.9362	0.9362	0.9895	0.9895	0.9362 ± 0.0000	0.9895 ± 0.0000	829.53 ± 3.85	0.0020
WOA-miRNA	0.9362	0.9362	0.9895	0.9895	0.9362 ± 0.0000	0.9895 ± 0.0000	4135 ± 344.00	0.0020
SSA-miRNA	0.9362	0.9362	0.9895	0.9895	0.9362 ± 0.0000	0.9895 ± 0.0000	765.96 ± 41.29	0.0020
AHALA-miRNA	0.9574	0.9574	0.9893	0.9733	0.9574 ± 0.0000	0.9733 ± 0.0000	676.19 ± 45.81	-

**Table 6:** Detailed accuracy by Class for our proposed AHALA-miRNA vs. another approach.

Method	Subtypes	TP Rate	FP Rate	Precision	Recall	F-Measure	Accuracy of Test Data
CFS_SVM [36]	LumA	0.78	0.09	0.82	0.78	0.80	0.7660
	LumB	0.50	0.07	0.62	0.50	0.56	
	H2	0.67	0.07	0.40	0.67	0.50	
	BL	0.83	0.00	1.00	0.83	0.91	
	Normal	1.00	0.05	0.83	1.00	0.91	
CFS_RF [36]	LumA	0.89	0.16	0.76	0.89	0.82	0.8058
	LumB	0.40	0.00	1.00	0.40	0.57	
	H2	0.67	0.02	0.67	0.67	0.67	
	BL	1.00	0.02	0.86	1.00	0.92	
	Normal	1.00	0.05	0.83	1.00	0.91	
AHALA-miRNA	LumA	1.00	0.06	0.94	1.00	0.95	0.9574
	LumB	0.89	0.02	0.89	1.00	0.94	
	H2	1.00	0.00	1.00	1.00	1.00	
	BL	0.88	0.00	1.00	0.88	0.94	
	Normal	1.00	0.00	1.00	0.00	1.00	

**Table 7:** Runtime and Accuracy Comparison of Optimization Algorithms.

Method	Avg. Runtime (s)	Accuracy (Mean ± Std)
Hill Climbing	27.69 ± 7.33	0.8894 ± 0.0519
ALA	1511 ± 98.39	0.9362 ± 0.0000
AHALA	676.19 ± 45.81	0.9574 ± 0.0000

Note: Runtimes are reported for optimizing neural network hyperparameters over 100 iterations in a single run. Accuracy is the average test accuracy over 10 runs on the TCGA miRNA dataset (231 samples, 208 miRNAs).

**Table 8:** Wilcoxon Signed-Rank Test Results.

Comparison	Statistic	*p*-Value
Hill Climbing vs. AHALA	0.00	0.0020
ALA vs. AHALA	0.00	0.0020

Note: Tests compare accuracy distributions over 10 runs. *p* < 0.05 indicates a significant difference.

**Table 9:** Cox Coefficient values for Selected miRNA genes.

miRNA	Coefficient	HR	HR_Lower	HR_Upper	*p*_Value	FDR
hsa.miR.135b.5p	−0.2468	0.7813	0.5726	1.0661	0.1196	0.2393
hsa.miR.93.5p	0.2637	1.3017	0.9621	1.7611	0.0874	0.2330
hsa.miR.134.5p	−0.2215	0.8013	0.6235	1.0300	0.0837	0.2330
hsa.miR.183.5p	0.0250	1.0253	0.8342	1.2603	0.8121	0.9689
hsa.miR.887.3p	0.2416	1.2732	0.9748	1.6630	0.0763	0.2330
hsa.miR.935	0.0752	1.0781	0.8026	1.4482	0.6174	0.9689
hsa.miR.3614.5p	0.0150	1.0151	0.7853	1.3121	0.9089	0.9689
hsa.miR.505.3p	−0.0061	0.9939	0.7322	1.3493	0.9689	0.9689

**Table 10:** Performance comparison of the AHALA-optimized neural network model on the TCGA test set and the independent GSE58606 dataset.

Dataset	Accuracy	Precision	Recall	F1 Score	AUC	MCC
TCGA Test	0.9574	0.9598	0.9574	0.9574	0.9893	0.9442
GSE58606	0.8297	0.8425	0.8297	0.8261	0.9497	0.7854

## Data Availability

The source code and datasets generated and/or analysed during the current study are available in the GitHub repository, https://github.com/guino001/HCALA_miRNA (accessed on 3 February 2026). Correspondence and requests for materials should be addressed to D.G.

## Supplementary Materials

The following supporting information can be downloaded at: https://www.mdpi.com/article/10.3390/cancers18040586/s1.
